# Programmable wavelength filter with double ring loaded MZI

**DOI:** 10.1038/s41598-021-04598-6

**Published:** 2022-01-27

**Authors:** Mi Wang, Xiangfeng Chen, Umar Khan, Wim Bogaerts

**Affiliations:** 1 Photonic Research Group, Department of Information Technology, Ghent University - IMEC, Ghent, Belgium; 2 Center of Nano and Biophotonics, Ghent University, Ghent, Belgium

**Keywords:** Integrated optics, Silicon photonics

## Abstract

We propose a novel filter circuit that incorporates a double ring resonator with a balanced Mach–Zehnder interferometer (MZI). The circuit has a response equivalent to a conventional ring loaded MZI filter, but with added flexibility in terms of configurability. The second-order filter can also be cascaded to realize higher-order filters. The circuit incorporates a two-stage input and output coupler to further reduce the effect of dispersion. A combination of local and global optimization strategies to program the filter, using tailored objective functions, have been tested in simulation and experiments. To our best knowledge, this is the first time a global optimization strategy is directly used in ARMA filter synthesis and simulation without any additional requirement. We further extend the optimization strategy into experiments and demonstrated its use in practical case for programmable filter circuits.

## Introduction

Wavelength filters are used to separate two wavelength bands into different output waveguides. They are basic building blocks for optical systems and have a great many applications in communication (wavelength division multiplexing), sensing or spectrometry^1–4^. When implemented as a waveguide circuit, optical filters are composed of couplers, delay lines, phase shifters, or ring resonators. These components are used to manipulate light to have constructive or destructive interference at certain wavelengths, such that a filter passband is generated. We can identify two basic classes of filters, i.e. finite impulse response (FIR) filter and infinite impulse response (IIR) filter. A FIR filter is usually composed by forward delays such as MZIs or arrayed waveguide gratings, and these are also called moving average (MA) filters. The IIR filter is composed by feedback loops such as ring resonators, and are also called autoregression (AR) filters. We hereby present a filter circuit which incorporates both MZI and double ring resonators, which has features from both IIR filter and FIR filter. Such filters, which combine both characteristics, are therefore called auto-regression/moving average (ARMA) filters. A typical example of such an ARMA filter is a so-called ring-loaded MZI, i.e. an MZI with a ring resonator in one or both arms^5^. The filter circuit we propose here extends the traditional ring loaded MZI by using a double (coupled) ring resonator that links the two arms of the MZI. This circuit has similar functionality as a ring loaded MZI with a single ring in each arm, but the additional coupling facilitates the configuration. We demonstrated this filter concepts both in simulation and experimentally, and developed optimization algorithms to configure the response of the filter.

The conventional ring loaded MZI filter was proposed by Madson^5^ more than 20 years ago. This design was theoretically demonstrated its capability to realize high-quality bandpass filters, and can theoretically fit certain exact bandpass profiles. Even though other types of structures have also been proposed to realize filters, such as nested ring Mach–Zehnder interferometers^6^, re-configurable silicon processors based on resonant self-coupled optical waveguides (SCOW)^7^, response shaping with a silicon ring resonator via double injection^8^, and the cross-ring resonator MZI interleavers^9^, none of them has demonstrated their structure to be able to exactly fit to the proposed bandpass filters.

We show that we can use our double ring-loaded MZI to fit different passband shapes. A hypercube sampling method is applied to generate near-random sampling of the parameter values to analyze pole-zero diagrams of the proposed design and compare it to a traditional ring-loaded MZI. Simulation results show that the two designs are equivalent in spectrum response, thus proving that our proposed design could also exactly realize bandpass filters. These simulations are detailed in the supplementary material.

Ideally, the tunable filter should be able to tune both the bandwidth and central wavelength at the same time. The common implementations of waveguide tunable filters are based on either coupled resonator optical waveguides (CROWs) or double-ring or quad-ring-loaded MZI. CROW filters have only poles, and therefore can only implement Butterworth or similar filters, and the circuit’s flexibility is limited due to the interdependency between the filter’s bandwidth and its out-of-band rejection^10, 11^. The ring-loaded MZI, which is demonstrated to be able to implement Butterworth, Chebyshev and elliptical filter functions, is theoretically proven to be tunable in both bandwidth and pass band shape. The recent experimental results of ARMA filters using a ring-loaded MZI by Sun^12^ demonstrated the tunability of such device, but the high in-band ripple and low roll-off make it hard to evaluate whether the device is configured to an elliptical filter or not , which is desirable for many applications. The automatically configured ring-loaded MZI by Choo^13^ successfully demonstrates that their configuration method could be used to configure the ring-loaded MZI to a elliptical filter with a bandwidth of 3–5 GHz with a small tunability range, but it lacks experimental results to demonstrate its performance as an interleaver or general-purpose optical filter, or its potential as a universal programmable filter. While such tuning procedure on one hand requires no expensive lab equipment, on the other hand the additional ring resonator and PDs introduce additional loss to the whole system. The experimental studies of programmable filters by Pérez^14^ also demonstrate some simple passband shapes such as single or coupled ring, and FIR filters such as MZIs with delay lines. In Table 1, we have made a performance summary and comparison of recent studies on tunable filters. Most of these utilize local optimization algorithms such as Nelder–Mead or particle swarm optimization algorithm, in this work, we have demonstrated the usage of the global optimization algorithm—basin-hopping—in filter synthesis.

**Table 1:** Summary and comparison of the integrated silicon photonic tunable filter performances.

	Tunability	Simulation	Demonstration	Optimization
^10^	Partially	5th Butterworth	Butterworth	Local
^11^	Fully	2nd Butterworth	Butterworth	Local
^13^	Fully	Butterworth	2nd, 4th	Assisted with
		Chebyshev, elliptical	Elliptical	Additional ring
^14^	Fully	Butterworth	FIR	Local
		Chebyshev, elliptical	Butterworth	
Our design	Fully	Butterworth	2nd elliptical	Local
		Chebyshev, elliptical	Chebyshev	Global

In this paper, we not only demonstrate the bandwidth tunability of our proposed ARMA filter (for both Chebyshev and elliptical filter spectra), but also demonstrate that our design can be tuned to a perfect elliptical filter both in simulation and experiments with a maximum 2 dB in-band ripple (which could be further optimized) and an overall 20 dB extinction ratio (and in few cases even 30 dB extinction ratio) for only a single-stage filter design. We also showed that our optimization algorithm helps to tune the parameters of the device accurately into elliptical and Chebyshev filters and our structure is a potential candidate for a universal programmable filter.

The remainder of the paper is structured as follows: In section “Filter circuit”, we describe the architecture of this filter circuit, and the effect of the tunable couplers. Section “Filter synthesis of an MZI loaded with serially coupled double ring” discusses the analytical transfer function and synthesis of the filter, and section “Cascade for higher order filter” shows how multiple filters can be cascaded into a higher-order filter. The numerical optimization algorithms to configure the filter are discussed in section “Optimization algorithm”. The experimental results on the fabricated chip are presented in section “Experimental result”. For the fabricated circuit, we extend the filter to use two-stage broadband tunable couplers^15^ at the input and output, to improve the dispersion effect of all output channels. Finally, we discuss the benefits and limitations of our filter circuit in section “Discussion”.

The supplementary materials contain the full mathematical derivation of the filter response, and a detailed comparison with the conventional ring-loaded MZI. We also present a tolerance analysis.

## Filter circuit

A ring resonator is a very useful building block for wavelength filters because it can provide a sharp resonance with a steep roll-off. However, a single ring resonator has its limitations for constructing pass-band filters with a box-like pass band. For that, more complex filter circuits are needed. Double ring resonators can provide a box-like passband with a limited bandwidth, and have been widely used in optical switches. Serially and parallel coupled ring resonator configurations have been described in detail in^16^. Ring resonators can also be combined with MZI filters: the ring loaded MZI structure^5^ has been theoretically demonstrated for realizing optical filters with optimum bandpass designs.

**Figure 1:**
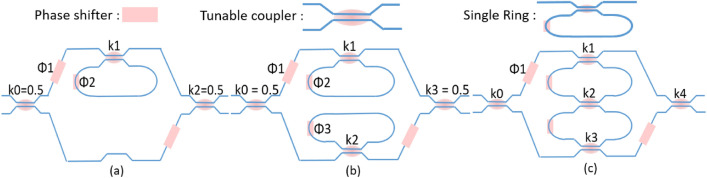
Schematic drawing of the single ring loaded MZI is shown in (**a**), schematic drawing of double ring loaded MZI is shown in (**b**), schematic drawing of coupled ring loaded MZI is shown in (**c**). The main difference between our design given in (**c**) and the ring loaded MZI given in (**b**) is that the two rings are connected with a tunable coupler, it is obvious that the design in (**c**) could be configured to a MZI with delay length of twice the ring circumference, which is not straightforward with the design in (**b**). The phase shifter is shown as a pink box, the tunable coupler is shown as a normal directional coupler with a pink dot in the middle, the single add drop ring is constructed by connecting the input and output port of the tunable coupler on the same side.

The optical filter circuit we propose here incorporates a double ring and a Mach–Zehnder interferometer. The schematic is presented in Fig. 1:

In the supplementary material, the *z* domain pole/zero diagram of the conventional ring loaded MZI and our design have been analyzed. We used a latin hypercube sampling method to explore the pole-zero relationship with the design parameters (the coupling coefficients and the phase shifts). Both designs can cover the available space in the *z* domain, which indicates their capability of exactly realizing optical filters with optimum band-pass designs.

## Filter synthesis of an MZI loaded with serially coupled double ring

We can describe the transmission of the filter from the input ports to the output ports using a $2\times 2$ transfer matrix. The transfer matrix can be calculated by multiplying the transfer matrices of each segment in the circuit: the input coupler, the double ring, and the output coupler. Because we choose to use ring resonators with a fixed length $L_{ring}$, we can apply the transfer matrix method (TMM) in the *z* domain, with $z=e^{j2\cdot n_{eff}(\lambda )\cdot L_{ring}/\lambda }$. The complex amplitudes of the input and output electromagnetic waves in the *z* domain have the following relationship between each other:

1 $$\begin{aligned} \begin{aligned} \begin{bmatrix} E_{out1}\\ E_{out2} \end{bmatrix}&= C_1\times P_1\times D_0\times P_0\times C_0 \times \begin{bmatrix} E_{in1}\\ E_{in2} \end{bmatrix} \end{aligned} \end{aligned}$$

where $E_{in1}$ and $E_{in2}$ are the complex amplitudes of the light in the fundamental waveguide modes at the inputs, $E_{out1}$ and $E_{out2}$ represent the same quantities at the outputs for the presented device. Matrix $C_i$ is the coupling matrix of each directional coupler, Matrix $P_i$ is the propagation matrix of a phase delay section (consisting of two parallel waveguides or phase shifters) and Matrix $D_i$ is the propagation matrix of the serially coupled double ring. The final transfer function in *z* notation can be written as the generic transfer function of a second-order filter:

2 $$\begin{aligned} \begin{aligned} H_{1}{z}&= \dfrac{b_{0}+b_{1} \cdot z^{-1} +b_{2} \cdot z^{-2}}{a_{0}+a_{1} \cdot z^{-1} + a_{2} \cdot z^{-2}} \end{aligned} \end{aligned}$$

where $a_i$ and $b_i$ are a function of the coupling ratios and phase shifts of the composition elements. In the supplementary material, we show that the $a_i$ and $b_i$ coefficients for the design in Fig. 1b are usually complex numbers while for the design in Fig. 1c these coefficients are real numbers. This circuit transfer function is then matched with the expression of a desired second-order filter with coefficients $a'_{0}, a'_{1}, \ldots , b'_{2}$, using least square fitting or Nelder-Mead method, where the error function for complex filer with $a_i$ and $b_i$ to be complex numbers is defined as following:

3 $$\begin{aligned} \begin{aligned} T&= ((\mathop {a_{0}} \cdot \mathop {a_{0}^{*}} - \mathop {{a'_{0}}^2})^2 + (\mathop {a_{1}} \cdot \mathop {a_{1}^{*}} - \mathop {{a'_{1}}^2})^2 + (\mathop {a_{2}} \cdot \mathop {a_{2}^{*}} - \mathop {{a'_{2}}^2})^2 + (\mathop {b_{0}} \cdot \mathop {b_{0}^{*}} - \mathop {{b'_{0}}^2})^2 \\&\quad + (\mathop {b_{1}} \cdot \mathop {b_{1}^{*}} - \mathop {{b'_{1}}^2})^2 + (\mathop {b_{2}} \cdot \mathop {b_{2}^{*}} - \mathop {{b'_{2}}^2})^2)^{1/2} \end{aligned} \end{aligned}$$

Likewise, the target function for real filter is:

4 $$\begin{aligned} \begin{aligned} T&= ((\mathop {a_{0}}^2 -\mathop {{a'_{0}}^2})^2 + (\mathop {a_{1}}^2 - \mathop {{a'_{1}}^2})^2 + (\mathop {a_{2}}^2 - \mathop {{a'_{2}}^2})^2 + (\mathop {b_{0}}^2- \mathop {{b'_{0}}^2})^2 + (\mathop {b_{1}}^2 - \mathop {{b'_{1}}^2})^2 + (\mathop {b_{2}}^2 - \mathop {{b'_{2}}^2})^2)^{1/2} \end{aligned} \end{aligned}$$

The full mathematical description of the fitting method has been provided in the supplementary material. Here, we will just explain how this fitting method works with the following example. A second-order Chebyshev type II filter is synthesized for the circuit in Fig. 1c. For a second-order low-pass Chebyshev type II filter with a stopband of 10 dB of magnitude response (20 dB for power response) and a normalized edge frequencies of 0.5 $\pi$ rad/sample, its transfer function is expressed as following:

5 $$\begin{aligned} \begin{aligned} H_{z}&= \dfrac{1-0.29 \cdot z^{-1} +0.27 \cdot z^{-2}}{0.37+0.25 \cdot z^{-1} +0.37 \cdot z^{-2}} \end{aligned} \end{aligned}$$

Now we equate the two transfer functions (2) and (5) together, and solve for the phase shifts and coupling values. The target function is given in Eq. (4). The minimization method in the Python package *scipy* is used to numerically solve the equations, which results in the following values:

6 $$\begin{aligned} \begin{aligned} \phi _1 = \pi , k_2 = 0.925, k_0 = k_4 = 0.146, k_1 = k_3 = 0.726 \end{aligned} \end{aligned}$$

**Figure 2:**
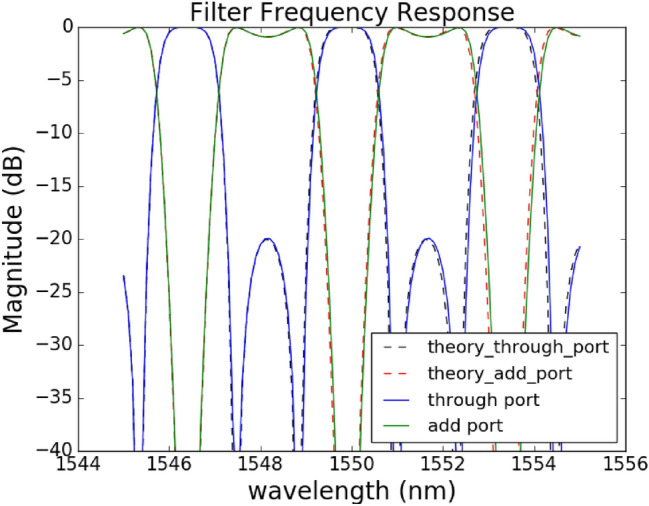
A comparison between the theoretical and Caphe simulated spectra for the through and add ports for an ideal Chebyshev type II filter.

We can see that no phase change in the ring is required in order to configure a second order filter. Such filter therefore has a different tuning strategy than the conventional ring loaded MZI where the phase offset of the two rings determines the frequency response. For these values, we find that the error function is zero, meaning we can obtain a perfect fit. The larger the error function, the worse the circuit performance would be compared to an ideal filter response. In Sect. 4 we will go through more details of how to tailor this error function. We have tested that the numerically solved equations have an error function of almost zero for the tested filters such as elliptical filter, Chebyshev type I and type II filters. The solved phase and coupling values were assigned to a circuit model built with the Caphe circuit simulator by Luceda Photonics^17^, the simulation result from Caphe are compared to the desired Chebyshev filter in Fig. 2, and we can see that the two spectrum responses overlap very well.

The proposed circuit in Fig. 1 has the following advantages: first, compared with the cascaded ring loaded MZI structure^18^, all the rings can be in resonance with each other, so, it would be more easy to tune the circuit in real-time experiments. Second, for microwave photonics, the proposed structure can realize more filter implementations, such as the coupled-resonator optical waveguide (CROW)^16^, the side-coupled integrated spaced sequences of optical resonators (SCISSOR) as well as ring loaded MZI^18^ and etc^9^, which makes the presented circuit a good candidate for a universal microwave programmable filter in a specialized microwave processor^19^. Finally, the proposed circuit can actually realize some pass band shapes that are not possible with a conventional ring loaded MZI design, which makes the proposed circuit more flexible in arbitrary waveform generation. The details are also discussed in the supplementary materials.

## Cascade for higher order filter

Higher-order filters provide more control over the frequency response with increasing order. The enhancement in roll-off with the higher order filters enables faster transition between the passband and stopband. To realize higher-order optical filters^18^, we choose the method of cascading first and second order filters to achieve the desired order, a method widely used in electronic filter design^20^. This technique can recognize any transmission zero and allows easy implementation. The drawback is the high sensitivity to variations in its components parameters^15^.

The order of a high-order filter can be either even or odd. For the synthesis of an *n* th even-order filter, *n*/2 second order filter can be cascaded to achieve the desired order. In the case for *n*th order odd filter, one biquadratic sections is replaced by one first-order section, if the general transfer function of a high order filter is given by:

7 $$\begin{aligned} \begin{aligned} H_{z}&= \dfrac{b_{m}z^{-m} + b_{m-1}z^{-(m-1)} + \cdots + b_1z^{-1} + b_0}{z^{-n} + a_{n-1}z^{-(n-1)} +\cdots + a_1z^{-1} + b_0} \end{aligned} \end{aligned}$$

where *m* is the order of enumerator and *n* is the order of the denominator, the even *n*th order filter can be expressed as following:

8 $$\begin{aligned} \begin{aligned} H(z) = \prod _{i=1}^{n/2} A_{\alpha _j}&= \prod _{i=1}^{n/2}\dfrac{b_{2i}z^{-2} + b_{1i}z^{-1} + b_{0i}}{z^{-2} + a_{1i}z^{-1} + a_{0i}} = \prod _{i=1}^{n/2} H_i(z) \end{aligned} \end{aligned}$$

The odd $n+1$th order filter will be expressed as following:

9 $$\begin{aligned} \begin{aligned} H(z) = \dfrac{b_{11}z^{-1}+b_{01}}{z^{-1}+a_{01}} \prod _{i=1}^{n/2} \dfrac{b_{2i}z^{-2} + b_{1i}z^{-1} + b_{0i}}{z^{-}2 + a_{1i}z^{-1} + a_{0i}} = H_1(z)\prod _{i=1}^{n/2} H_i(z) \end{aligned} \end{aligned}$$

Each first and second order equation can be designed independently and individually. The main drawback in this method is the difficulty in tuning the component variation without assistance of a feedback loop.

**Figure 3:**
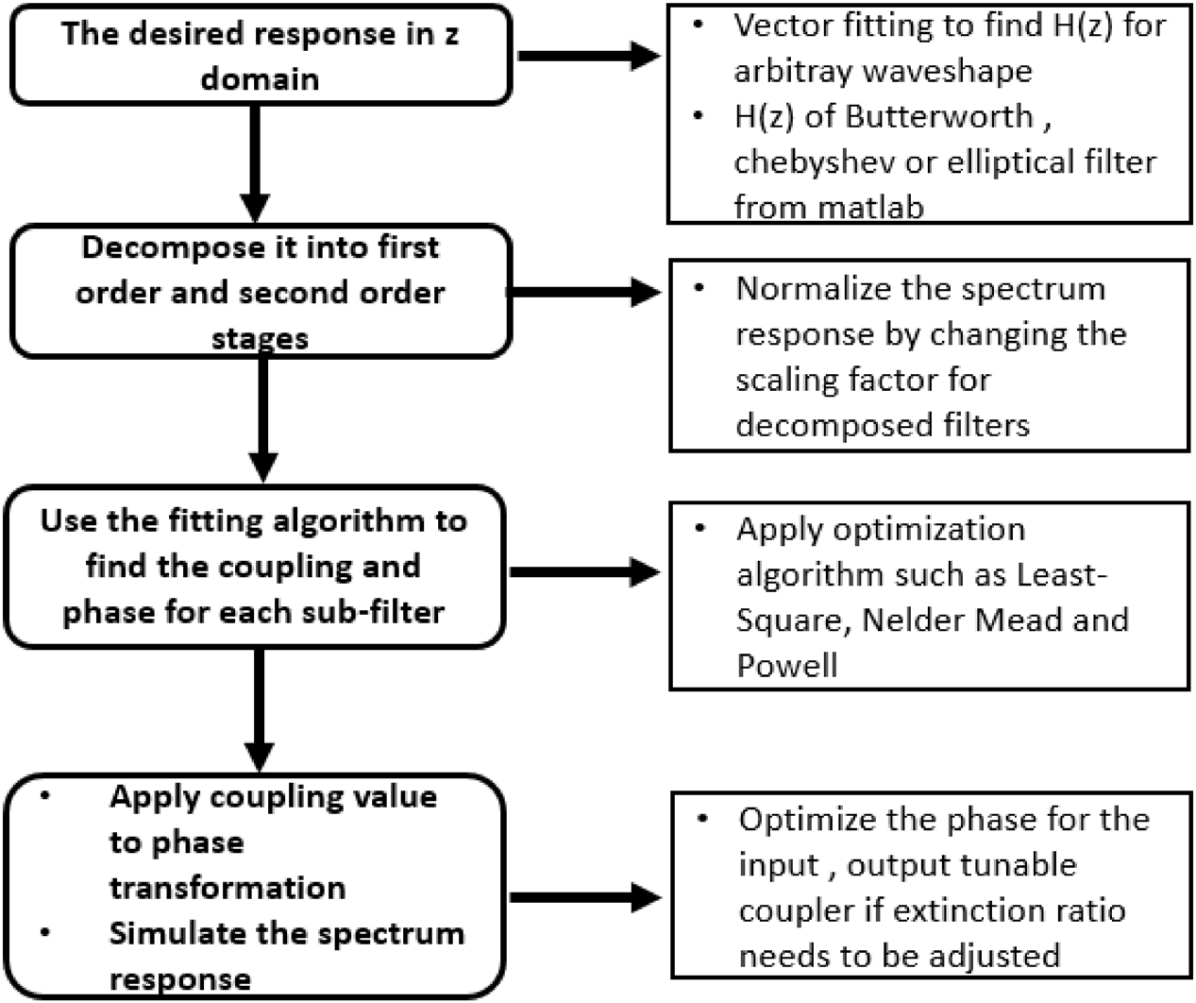
Workflow for arbitrary shape or higher order filter synthesis.

In this section, we will generate a fourth-order filter with the proposed optical circuit using this cascade technique. The workflow of the synthesis method for a higher-order filter is presented in Fig. 3.

We now show an example for the filter synthesis with a fourth-order elliptical low-pass filter with normalized edge frequencies of 0.5$\pi$ rad/sample, 2 dB pass band ripple and 40 dB attenuation^21^. The transfer function for such a filter is written as:

10 $$\begin{aligned} \begin{aligned} H_{z}&= \dfrac{0.2318+0.3378 \cdot z^{-1}+0.5297 \cdot z^{-2}+0.3378\cdot z^{-3}+0.2318\cdot z^{-4}}{1-0.3396\cdot z^{-1}+1.2275\cdot z^{-2}-0.3118\cdot z^{-3}+0.2964\cdot z^{-4}} \end{aligned} \end{aligned}$$

*H*(*z*) can be decomposed into two second-order filters, $H_{1}(z)$ and $H_{2}(z)$, such filter decomposition is done easily in Matlab using the function *zp2tf*, and the corresponding transfer functions are:

11 $$\begin{aligned} \begin{aligned} H_{1}(z)&= \dfrac{0.39+0.0910\cdot z^{-1}+0.39\cdot z^{-2}}{1+0.0.0094\cdot z^{-1}+0.9023\cdot z^{-2}} \end{aligned} \begin{aligned} H_{2}(z)&= \dfrac{0.3+0.3673\cdot z^{-1}+0.3\cdot z^{-2}}{1-0.3490\cdot z^{-1}+0.0785\cdot z^{-2}} \end{aligned} \end{aligned}$$

**Figure 4:**
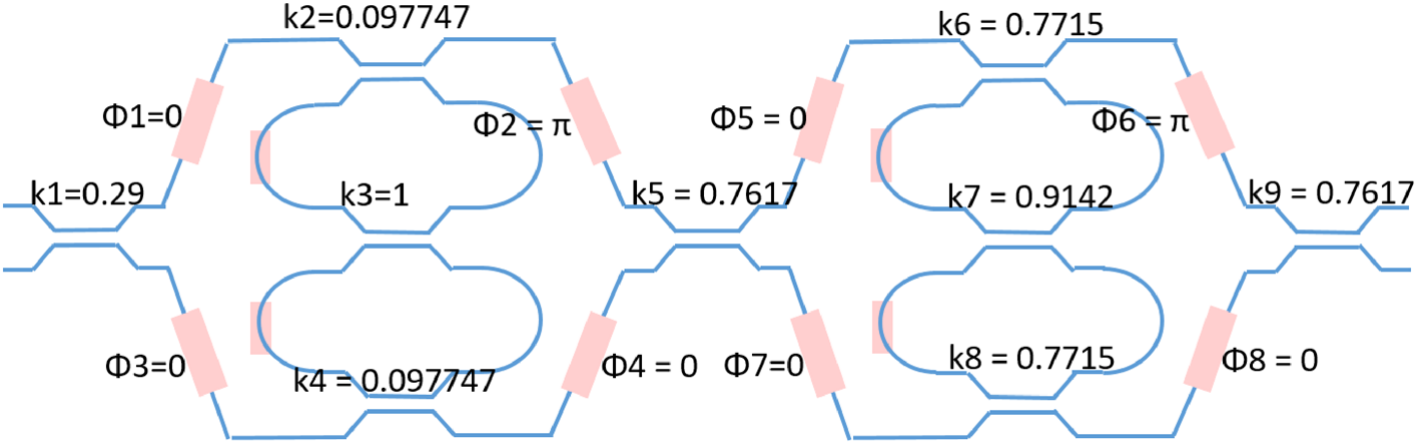
Schematic drawing of the programmable filter and the corresponding coupling and phase values to realize the designed elliptical filter.

After executing our before mentioned fitting algorithm for each stage, we get the desired coupling value for the cascaded filter design in Fig. 4.

**Figure 5:**
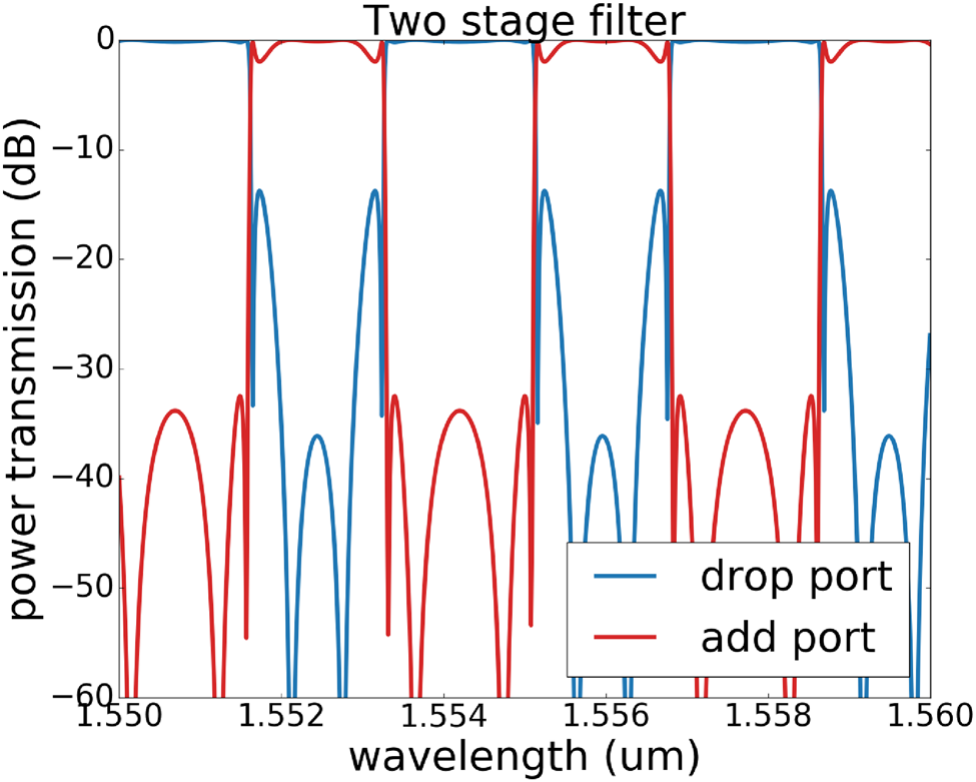
Spectrum response of the elliptical filter generated with corresponding Caphe model for the circuit in Fig. 4.

We then generate the corresponding Caphe model for the circuit in Fig. 4 and we can see the corresponding spectrum response in Fig. 5. The resulting filter has around 34 dB extinction ratio as shown in Fig. 5, which is slightly lower than the targeted 40 dB response.

We have demonstrated that our double ring loaded MZI can implement an elliptical filter with the given filter synthesis and optimization method. The circuit is also capable of implementing filter architectures as shown in^9, 13, 18^. In the supplementary material, we have shown another example to realize a Chebyshev type II filter.

## Optimization algorithm

There are still several challenges related to our synthesis and optimization procedure. As a starting point, the coupling coefficients corresponding to elliptical filters have been calculated for the lossless case. However, if we want to include the actual waveguide and coupler losses in our model, we could either insert a loss factor in our analytical derivation, or we could solve the problem by treating the loss as a perturbation in our optimization algorithm. When we take the actual behaviour of the components into account, the spectral dispersion induced by the wavelength dependence of the couplers, fabrication variations, and thermal and electronic cross talk, could all be compensated when the optimization algorithm is applied^10, 22^.

The quality of the optimization depends on the target function, and which features of the filter transmission spectrum are the most relevant. We define the target function as follows: The error (difference) between the optimization result and the desired filter response is denoted as $x_{lin}$, and the difference on a dB scale is denoted as $x_{dB}$. The first emphasises deviations in the pass band, while the second emphasises the rejection band. The target function is a weighted combination of both:

12 $$\begin{aligned} \begin{aligned} T = w_{1} \cdot x_{lin} + w_{2} \cdot x_{dB} \end{aligned} \end{aligned}$$

As wavelength filters are phase-sensitive interference-based circuits, we expect that the optimization space has many local optima. It is therefore useful to look into various classes of optimization algorithms that could be helpful for our problem. In this section we focus on two kinds of algorithms, and we show that these algorithms are sufficiently robust to solve our problem in simulation and eventually can be incorporated to optimize and tune the experimental filter circuits in real time.

### Local optimization: Nelder–Mead and Powell

*Nelder–Mead*^23^ and *Powell*^24^ are two free-derivative optimization methods. Both methods work well for local optimization starting from a good initial estimate. The *Nelder–Mead* is slow and has a convergence order of 1, which means that large termination errors may occur due to limited iteration steps. It has been tested that the *Powell* method converges much faster than *Nelder–Mead* method in our experiments. The *Nelder–Mead* is often used when the number of optimizable parameters is very large.

**Figure 6:**
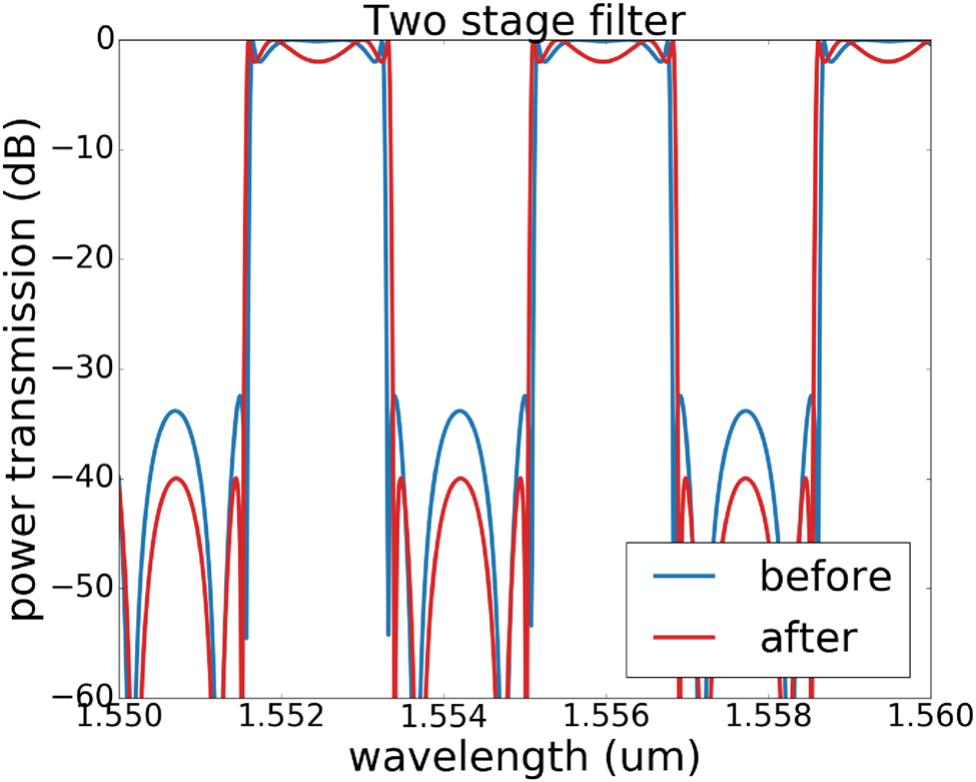
Spectrum response of an optimized elliptical filter targeting 40 dB extinction ratio.

**Case one**: The elliptical filter design in Fig. 4 has an extinction ratio of around 34 dB. We could use the Nelder–Mead method to further optimize the spectral response. In this optimization problem, the target function is defined as the error between the spectrum response of the Caphe model on a dB scale and the 40 dB elliptical filter response. The circuit simulation in Caphe^17^ with an initial 34 dB extinction ratio is optimized to the desired 40 dB elliptical filter response and the result is shown in Fig. 6.

**Figure 7:**
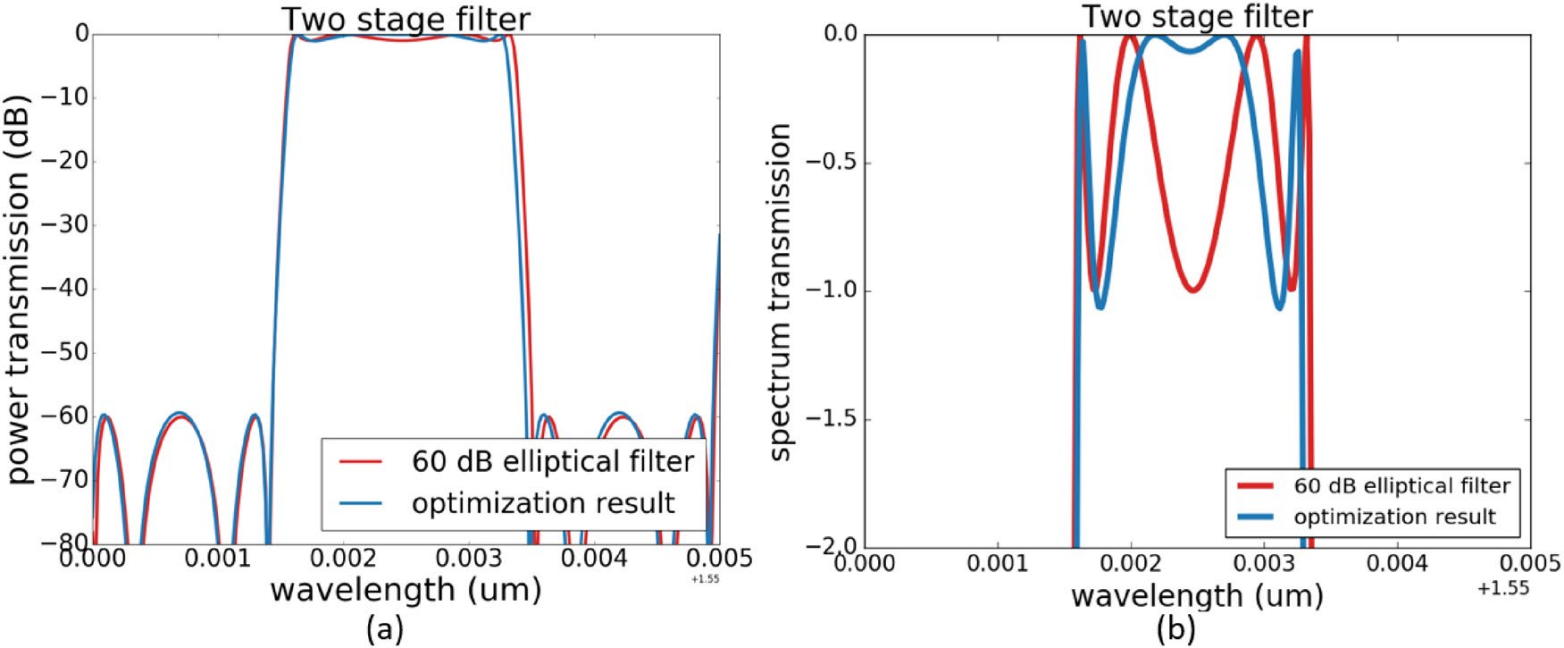
Spectrum response of the optimized elliptical filter for an elliptical filter with 1 dB in band ripple and 60 dB extinction ratio. (**a**) is the final optimization result, which has the desired 60 dB extinction ratio. (**b**) is a zoom-in on the pass band ripple.The second optimization step successfully lowered this in-band ripple.

**Case two**: This second example illustrates more clearly how to define the target function. This time, we start from the 40 dB elliptical filter with 2 dB in-band ripple and try to optimize it further to a 60 dB filter with 1 dB in-band ripple. We use multiple optimization steps this time. In the first optimization step, the target function is the error between the 60 dB elliptical function with 1 dB in-band ripple and our original circuit response ( the 38 dB elliptical filter with 2 dB in-band ripple ). After optimization, we clearly see that the optimized result now has a extinction ratio of 60 dB, but the in-band ripple is larger than expected. In the next optimization steps, we focus on reducing the in-band ripple by assigning a larger weight $w_{1}$ to $x_{1}$. The final optimization result is shown in Fig. 7.

### Global optimization: basin hopping

As an alternative optimization method, we utilized a global optimization algorithm—*basin hopping*^25^. Basin hopping is a two-phase method that combines a global stepping algorithm with local minimization. We still use the Nelder–Mead method for the local minimization phase. The number of basin-hopping iterations is set according to the difficulty of the problem.

**Figure 8:**
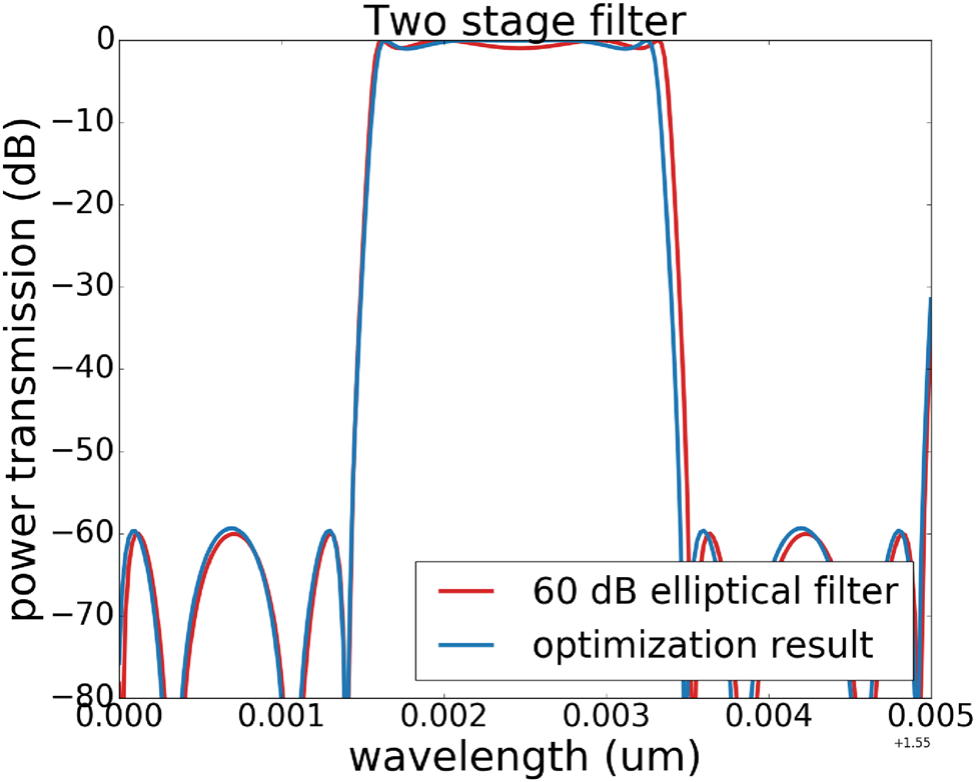
Spectrum response of the optimized elliptical filter pass band with basin-hopping optimization. A 60 dB elliptical filter is the targeted filter response, and the optimization starts from a random position. The final optimized result overlaps very well with the targeted filter response.

In our experiment of starting from a random position and optimizing it to an elliptical filter, 10 basin-hopping iterations and 100 Nelder–Mead evaluations for each local optimization is used. If we already have a good guess about the coupling values for the filter design (for example, we set $k3 = 1, k2 = k4$ and $k6 = k8$ for the elliptical filter design in Fig. 4), the optimization went very smoothly. However, if we do not have any constraint on the initial coupling values for the design in Fig. 4, we need more optimization steps and even need to adjust the target function to get the best optimization result. One typical target function in our experiments is defined as $T = 1\cdot x_{1} + 100\cdot x_{2}$. The final optimized result is shown in Fig. 8.

## Experimental result

In this section, the experimental results for the programmable filter are presented. The silicon photonics chip is fabricated using the process design kit (PDK) for IMEC’s iSiPP50G process for the standard building blocks, augmented with qualified components developed in our group. For instance, the phase shifters are parametric cells that generate doped silicon resistors close to the waveguide. The mask layout and the corresponding microscopic image are shown in Fig. 10. The MZI is loaded with two sets of double ring design with the input and output tunable coupler designed as a two-stage cascaded MZI, as such a design has been proven to have more broadband response^15^. We expect that this will help us to improve the uniformity of the extinction ratio for different channels since the dispersion of the filter response is mainly caused by the dispersion of the tunable coupler.

**Figure 9:**
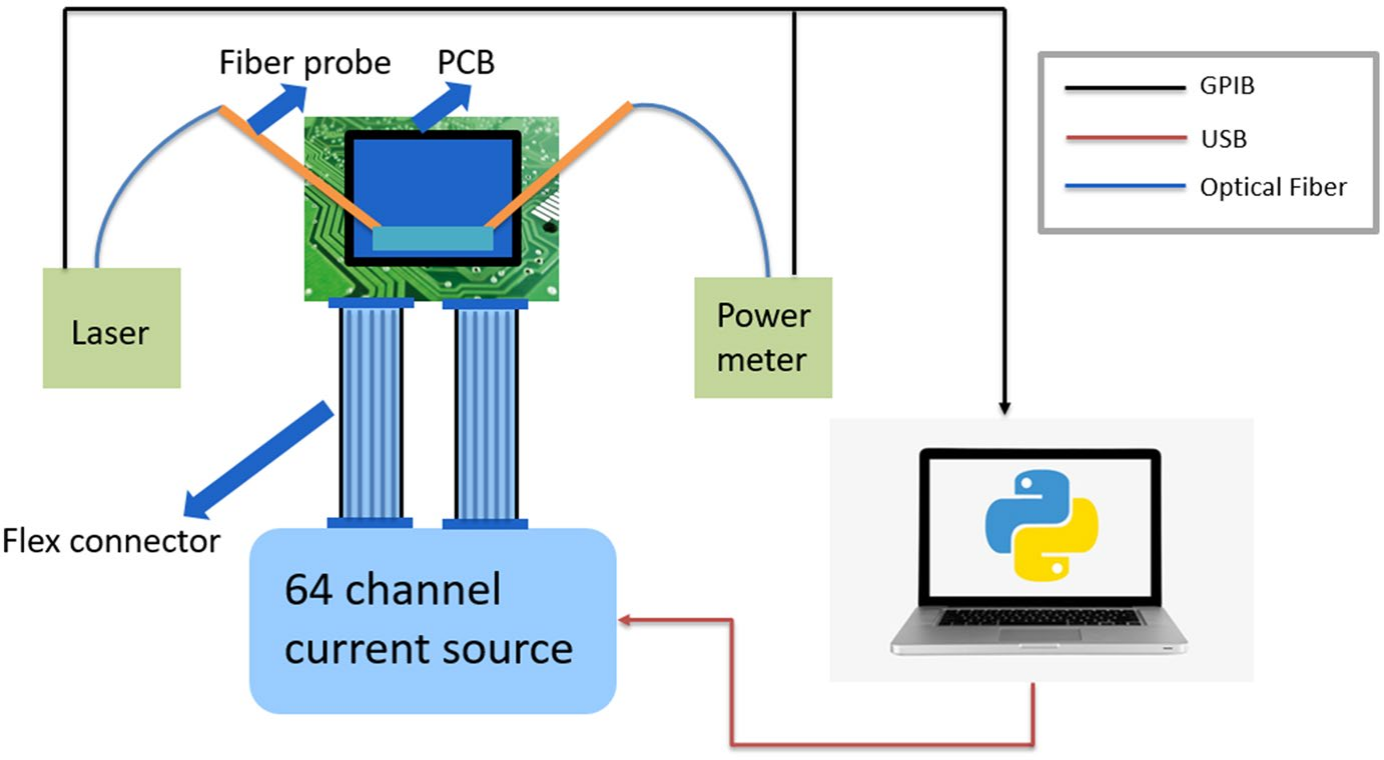
The measurement setup is composed of a tunable laser as input light source, a power meter to measure the spectrum response for the drop and through port and a 64 channel current source to control the heaters. All these measurement instruments are controlled by the computer, and the optimization algorithm takes the real-time measured power values as input and controls the current source.

The measurement setup is shown in Fig. 9. The measurement process contains the following steps: (1) the target analog or digital filter is chosen, for example a second-order low-pass Chebyshev type II filter with a stopband of 10 dB of magnitude response (20 dB for power response) and a normalized edge frequencies of 0.5 $\pi$ rad/sample is chosen as the target filter. Then the filter synthesis procedure is implemented in order to calculate the phase responses of the phase shifters for our filter design. (2) We then map the phase responses of the phase shifters to the voltage or current that we want to apply. This step is called calibration of the phase shifter. Normally we have a asymmetrical MZI as our test design for the calibration. (3) If the thermal crosstalk or electrical crosstalk is insignificant, then the initial spectrum response is similar to the targeted filter response. However, the electric crosstalk is high in our digital-to-analog converters (DAC), thus the initial result is severely disturbed compared to the ideal response, however the initial voltage values are still a good starting point for our optimization process. (4) The optimization algorithm is chosen, the voltage of the heaters are set as the parameters for optimization, the target function defined as the difference between the target filter response and the measured response is being minimized in this process.

The experimental result is obtained first by assigning the phase shifters with the calculated phase values (see the supplementary material for the programmable filter design), however the crosstalk of different electronic channels of the digital-to-analog convertor and thermal crosstalk of the phase shifters degrades the spectrum responses severely compared to the original prediction. Therefore the optimization algorithm is needed to finetune the device. The *Powell* method is chosen for this step since it converges faster than the *Nelder–Mead Method* in experimental settings.

**Figure 10:**
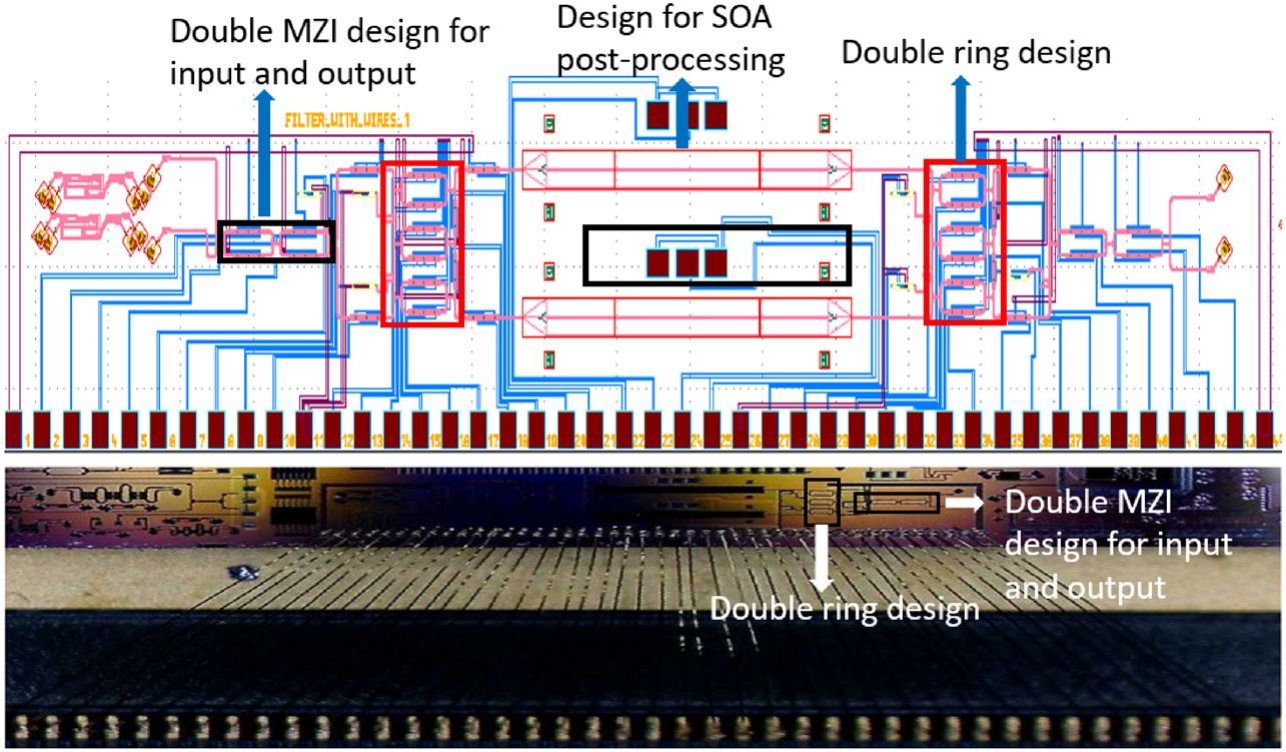
Fabricated double-ring-loaded MZI filter circuit. The upper figure shows the mask layout of the fabricated chip. The basic design is a MZI loaded with two sets of double rings, where the input and output coupler for the MZI are a double-stage balanced MZI^15^. This design also incorporates a placeholder for later semiconductor optical amplifier (SOA) post-processing using transfer printing^26^. Without SOA, the placeholder introduces additional insertion losses. Monitor photodiodes are connected to the inside of the ring resonators and in the arms of the MZIs. The lower image is a microscopic image of the chip, the chip is wire-bonded to a PCB board for electronic control.

**Figure 11:**
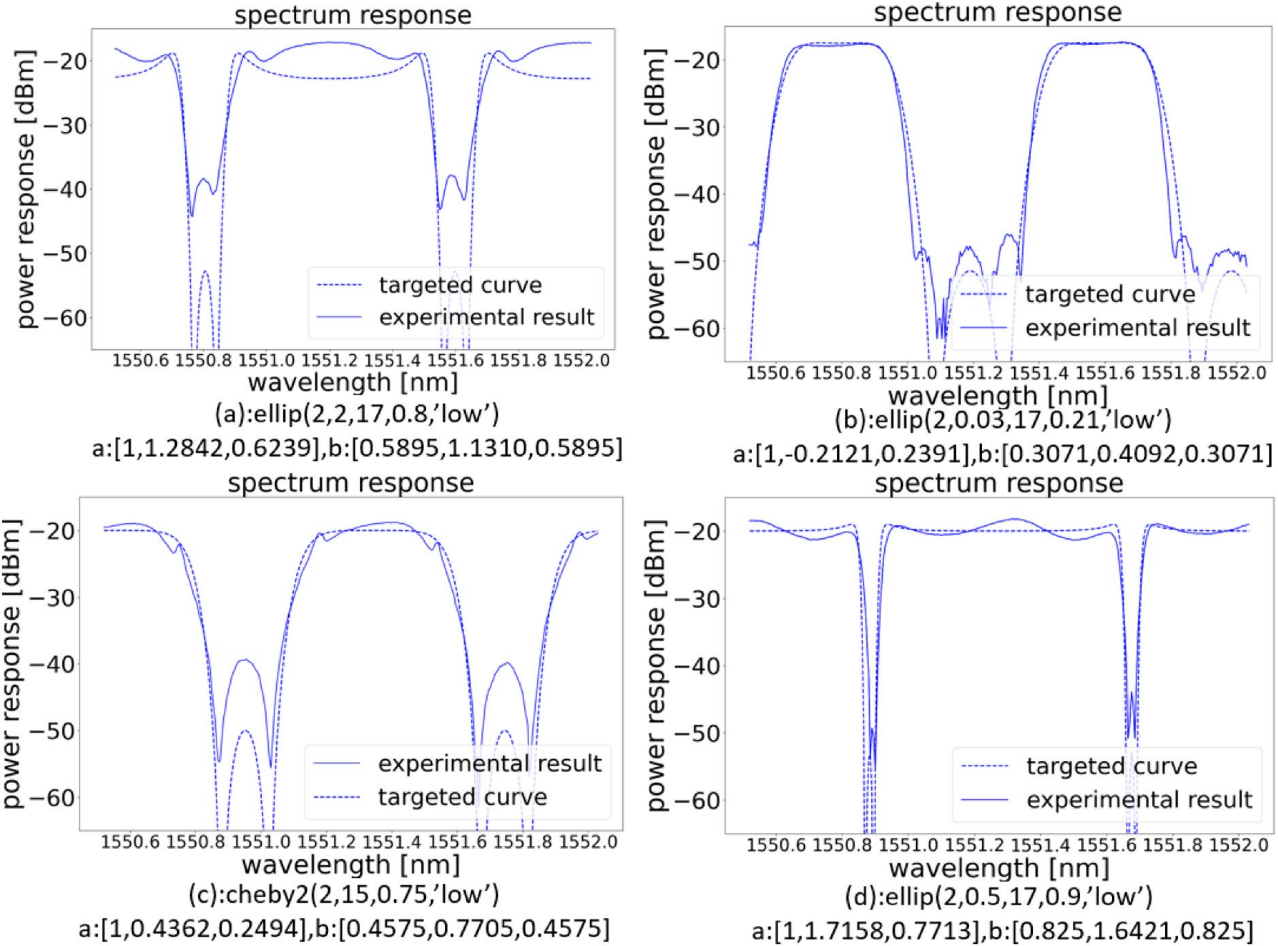
Experimental transmission spectra of the double ring-loaded MZI filter. Each plot shows the target design (with the $a_i$ and $b_i$ coefficients listed below the plot). The experimental results are obtained by optimizing the spectral response from the chip outputs by driving the on-chip actuators in real time. From the measurement we can see, the experimental results matches the simulation very well, and the overall extinction ratio exceeds 20 dB for all designs.

**Figure 12:**
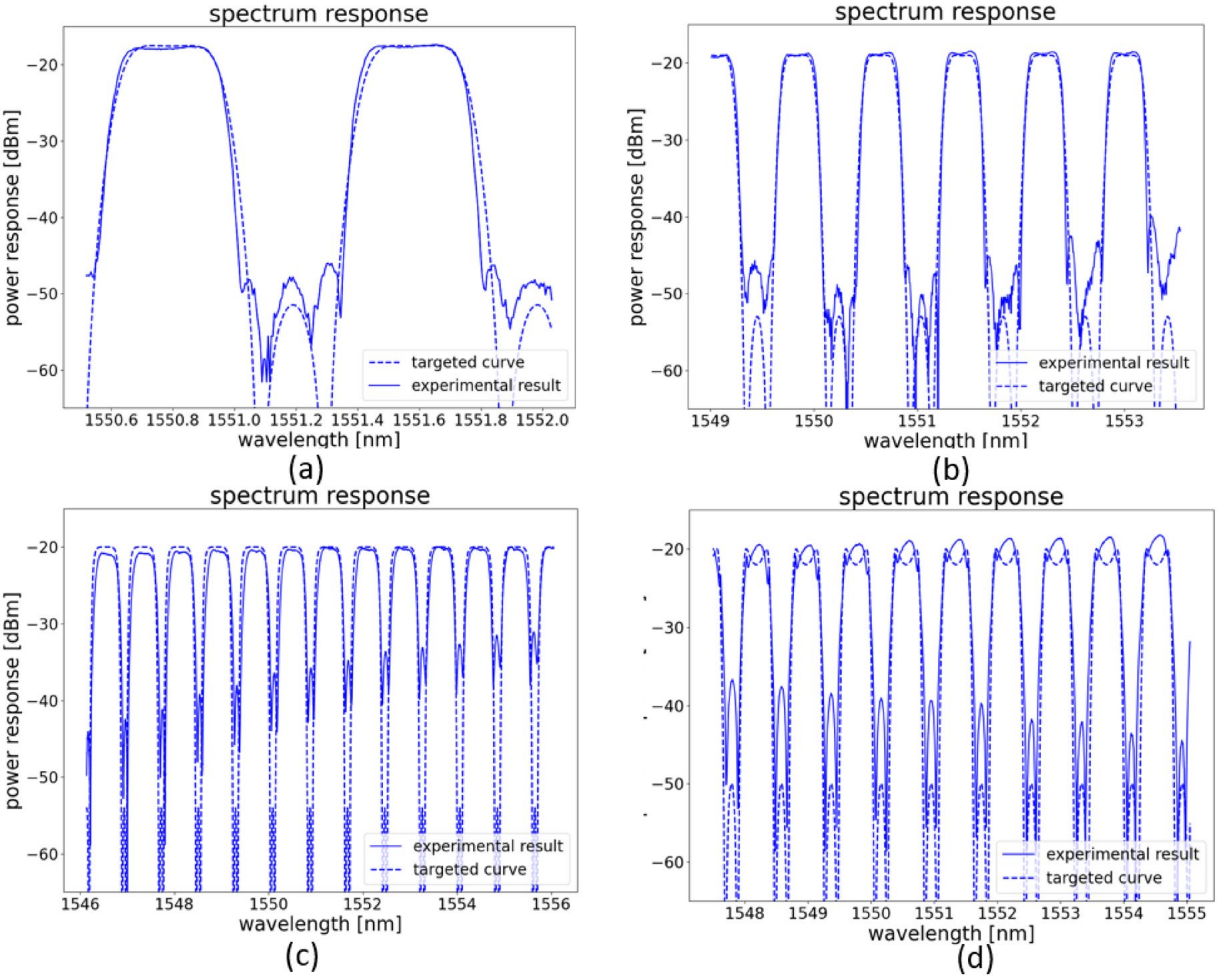
Effect of dispersion effect for different filter configurations. (**a**) is a re-plot of Fig. 11b, the wavelength range is from 1550.6 to 1552.0 nm. (**b**) is the same filter measured in a larger wavelength range of 1549–1554 nm. (**c**) and (**d**) are two examples with more than 10 channels in 10 nm wavelength range. The dispersion effect has a strong impact on the extinction ratio between different channels.

Figure 11 shows that the measured transmission matches well with the targeted simulation curve, the extinction ratio of 20 dB is achieved by all the filter types, some even have an extinction ratio of 30 dB. The corresponding coupling matrix for designed filters in Fig. 11 is given in Table. 2. The spectrum response in a larger wavelength range is also measured in Fig. 12 , and we see (as expected) that the dispersion of the building blocks starts to have a strong effect on the extinction ratio. Fig. 12a is filter response in a small wavelength range, while Fig. 12b is the same filter response measured in a larger wavelength range, among the measured 5 channels, only 2 channels have an extinction ratio over 30 dB, however all the channels have an extinction ratio over 20 dB. Figure 12c, d shows the filter response over 10 channels, where the dispersion effect is even more obvious.

**Table 2:** This table contains coupling matrix for designed filters in Fig. 11.

Design	Coupling: $k_{0}$, $k_{1}$, $k_{2}$, $k_{3}$, $k_{4}$	Phase: $\Phi _{1}$
(*a*)	0.16005768, 0.43616947, 0.32919224, 0.30963887, 0.82755031	$\pi$
(*b*)	0.60552951, 0.76092306, 0.95295684, 0.76092306, 0	0
(*c*)	0.0, 0.758446465, 0.794976, 0.758446465, 0.144481524	$\pi$
(*d*)	0.34447773, 0.1678724, 0.04629151, 0.28641485, 0.68461482	$\pi$

The coupling values and phase are calculated for the schematic drawing shown in Fig. 1c.

## Discussion

In this manuscript, we propose a novel architecture of a programmable ring loaded MZI filter to implement a programmable filter. The FSR of the measured filter is 0.785 nm, and it is designed for 100 GHz channel spacing. Such a filter has the potential to be used as an optical interleaver. With the accompanying optimization strategy we have shown that we can configure different ARMA filter transmission spectra. We did this by only tuning the coupling coefficients of the tunable couplers in the filter, without tuning the phase shifters.The tuning strategy proposed by Gihoon^13^ for the conventional ring-loaded MZI is enabled by one additional ring with photodiode and by characterizing each individual tunable coupler. Such an algorithm would work if the thermal and electric crosstalk between heaters is sufficiently small. However, due to the large thermal and electric crosstalk in our system, the initial calibration did not yield a good performance. Thus another optimization step for multiple heaters together is necessary.

In our design, the photodiode inside the ring could be used to acurately calibrate the coupler values and also to align the two rings. It can be used in the coarse tuning step, and then the optimization algorithm could be applied in a second step for fine tuning. One important factor why such tuning algorithm would work in our filter is because of the design itself—the rings are initially zero-phase and in phase with each other (see supplementary material), and drift during operation could therefore be easily calibrated with the help of an integrated photo-diode. The optimization algorithm thus only deals with the coupler values, which made the whole tuning process much faster. The temperature change of the phase shifters over time can also be compensated with the photodiodes once the initial calibration has been performed. The drawback of including monitor diodes in the filter circuit is that these induce losses. Especially when monitor diodes are introduced in the rings, this limits the quality factor, and therefore the promixity of the poles to the unit circle in the *z* plane. Indeed, in our circuit the losses are mainly induced by the monitors, the monitors tap off 1% of the light in the waveguide. Some losses in the filter could be compensated by integrating amplifiers inside or outside the filter (which was foreseen in the design, as shown in Fig. 10a. Configuration difficulties due to thermal crosstalk could be reduced by using heaters with better insulation^27^, or use a non-dissipative tuning mechanism such as MEMS^28^.

In our experiment, the tunable couplers in the rings did not have sufficient range to cover the entire 0–100% coupling range due to fabrication variations in the 50:50 directional couplers. We still see that in experiments the default cross state for the second set of two coupled rings (three couplers of the second set of double ring structures) does not impact the performance of the first set of coupled rings. Besides tuning the device with the real time optimization strategy, an alternative way of using such a filter circuit would be to build a look-up table for the filter, and once the filter is calibrated for a certain performance, the tuning parameters would be recorded. Such a configuration strategy works well even without monitors.

The estimated Q factor is 18184, the full calculation of it is in the supplementary material. The main loss in our system is from the loss of the waveguide and the doped heater. If we change the platform to *SiN* platform, we could further improve the Q factor of our system.

The power consumption of the filter depends on the number of heaters used in the system. For a single stage of a coupled ring-loaded MZI we require 10 phase shifters, and we’re not using the other actuators in the circuit in Fig. 10. For full tunability, we can assume that each phase shifter’s average shift is $\pi$, with an averaged power consumption of 20 mW. Thus, the total power consumption in our current system is around 200 mW. We are considering to replace the doped heater with undercut heater in our future design, the power consumption of the undercut heater is around 1.5 mW/$\pi$ reported in literature^27^.

## Conclusion

In this manuscript, we have proposed a ring-resonator based circuit to realize a configurable second-order autoregressive-moving-average (ARMA) filter. We have shown that such circuit can be cascaded to realize higher-order filters. We also demonstrated optimization of the tuning coefficients both in simulation and in experiments.

## Supplementary Information

Supplementary Information.
