# Supplementary material for: Programmable wavelength filter with double ring loaded MZI
Source: Sci Rep. 2022 Jan 27;12:1482. doi: 10.1038/s41598-021-04598-6 (PMC8795437; doi:10.1038/s41598-021-04598-6)
Supplement: Supplementary file 1 — Supplementary Information. [file 41598_2021_4598_MOESM1_ESM.pdf]

Programmable filter design with double ring loaded MZI

1. FILTER SYNTHESIS OF AN MZI LOADED WITH SERIALLY COUPLED DOUBLE RING

The transmission of the filter from the input ports to the output ports can be calculated by the following transfer matrix multiplication in the z domain. Because we choose to use ring resonators with a fixed length L_{ring}, we can apply the transfer matrix method (TMM) in the z domain, with $z = e^{j2.n_{eff}(\lambda).L_{ring}/\lambda}$.

The complex amplitude of the input and output electromagnetic waves in the z domain have the following relationship between each other:

$$\begin{bmatrix} E_{out1} \\ E_{out2} \end{bmatrix} = C_1 \times P_1 \times D_0 \times P_0 \times C_0 \times \begin{bmatrix} E_{in1} \\ E_{in2} \end{bmatrix} \tag{S1}$$

The coupling matrix, C_i, is given by:

$$C_i = \begin{bmatrix} \sqrt{1-k_i} & -j\sqrt{k_i} \\ -j\sqrt{k_i} & \sqrt{1-k_i} \end{bmatrix} \tag{S2}$$

where k_i is the power cross-coupling coefficient, and $1 - k_i$ is the straight-through coefficient for the directional coupler (assuming lossless operation). The propagation matrix P_i of each phase delay section is given by:

$$P_i = \begin{bmatrix} e^{-j\phi_i} & 0 \\ 0 & 1 \end{bmatrix}, \tag{S3}$$

where we assume no loss, and equal length for waveguide propagation, so the common propagation terms can be ignored.

The propagation matrix D_i of serially coupled double ring section is given by:

$$D_i = \frac{1}{\delta} \begin{bmatrix} X_1 & -j \cdot e^{-1j(\phi_2+\phi_3)} \cdot S_1 \cdot S_2 \cdot S_3/z \\ -j \cdot e^{-1j(\phi_2+\phi_3)} \cdot S_1 \cdot S_2 \cdot S_3/z & X_2 \end{bmatrix}, \tag{S4}$$

where δ is written as:

$$\begin{aligned} \delta = 1 - e^{-2j\cdot\phi_2} \cdot C_1 \cdot C_2/z - e^{-2j\cdot\phi_3} \cdot C_2 \cdot C_3/z + \\ e^{-2j\cdot(\phi_2+\phi_3)} \cdot C_1 \cdot C_3 \cdot (1 - C_2^2)/z^2 + e^{-2j\cdot(\phi_2+\phi_3)} \cdot C_1 \cdot C_2^2 \cdot C_3/z^2 \end{aligned} \tag{S5}$$

And X_1 and X_2 are written as:

$$\begin{aligned} X_1 = (C_1 \cdot z^2 + (-C_2 \cdot e^{-2j\cdot\phi_2} - C_1 \cdot C_2 \cdot C_3 \cdot e^{-2j\cdot\phi_3}) \cdot z + C_3 \cdot e^{-2j\cdot\phi_2 - 2j\cdot\phi_3} - \\ C_2^2 \cdot C_3 \cdot e^{-2j\cdot\phi_2-2j\cdot\phi_3} + C_1^2 \cdot C_2^2 \cdot C_3 \cdot e^{-2j\cdot\phi_2-2j\cdot\phi_3} + \\ C_2^2 \cdot C_3 \cdot e^{-2j\cdot\phi_2} \cdot e^{-2j\cdot\phi_3} - C_1^2 \cdot C_2^2 \cdot C_3 \cdot e^{-2j\cdot\phi_2} \cdot e^{-2j\cdot\phi_3})/z^2 \end{aligned} \tag{S6}$$

$$\begin{aligned} X_2 = (C_3 \cdot z^2 + (-C_2 \cdot e^{-2j\cdot\phi_3} - C_1 \cdot C_2 \cdot C_3 \cdot e^{-2j\cdot\phi_2}) \cdot z + C_1 \cdot e^{-2j\cdot\phi_2 - 2j\cdot\phi_3} - \\ C_2^2 \cdot C_1 \cdot e^{-2j\cdot\phi_2-2j\cdot\phi_3} + C_1^2 \cdot C_2^2 \cdot C_3 \cdot e^{-2j\cdot\phi_2-2j\cdot\phi_3} + \\ C_2^2 \cdot C_1 \cdot e^{-2j\cdot\phi_2} \cdot e^{-2j\cdot\phi_3} - C_2^2 \cdot C_3^2 \cdot C_1 \cdot e^{-2j\cdot\phi_2} \cdot e^{-2j\cdot\phi_3})/z^2 \end{aligned} \tag{S7}$$

The final transfer function in the z notation can be written as the generic transfer function of a second-order filter:

$$H_1 z = \frac{b_0 + b_1 \cdot z^{-1} + b_2 \cdot z^{-2}}{a_0 + a_1 \cdot z^{-1} + a_2 \cdot z^{-2}} \tag{S8}$$

where a_i and b_i are a function of the coupling coefficients and phase shifts. The coefficients of the transfer function in Eq. S8 are given by:

$$b_0 = C_0 C_1 C_4 e^{1j\phi_1 + 6j\phi_2 + 6j\phi_3} + C_3 S_0 S_4 e^{4j\phi_2 + 4j\phi_3} \tag{S9}$$

$$\begin{aligned} b_1 = & C_2 S_0 S_4 e^{2j\phi_2} \cdot e^{2j(\phi_2+\phi_3)} - C_0 C_2 C_4 e^{1j\phi_1} * e^{2j\phi_3} * e^{2j(\phi_2+\phi_3)} + \\ & C_1 C_2 C_3 S_0 S_4 e^{2j\phi_3} e^{2j\phi_2 + 2j\phi_3} - C_0 C_1 C_2 C_3 C_4 e^{1j\phi_1} e^{2j\phi_2} e^{2j(\phi_2+\phi_3)} + \\ & C_0 S_1 S_2 S_3 S_4 e^{2j(\phi_2+\phi_3)} e^{1j(\phi_2+\phi_3)} + C_4 S_0 S_1 S_2 S_3 e^{2j(\phi_2+\phi_3)} e^{1j(\phi_1+\phi_2+\phi_3)} \end{aligned} \tag{S10}$$

$$\begin{aligned} b_2 = & C_1 C_2^2 S_0 S_4 e^{2j(\phi_2+\phi_3)} - C_1 S_0 S_4 e^{2j(\phi_2+\phi_3)} - C_1 C_2^2 S_0 S_4 e^{2j(\phi_2+\phi_3)} + \\ & C_0 C_2^2 C_3 C_4 e^{1j\phi_1} e^{2j(\phi_2+\phi_3)} + C_0 C_3 C_4 e^{1j\phi_1} e^{2j(\phi_2+\phi_3)} + C_1 C_2^2 C_3^2 S_0 S_4 e^{2j(\phi_2+\phi_3)} - \\ & C_0 C_1^2 C_2^2 C_3 C_4 e^{1j\phi_1} e^{2j(\phi_2+\phi_3)} - C_0 C_2^2 C_3 C_4 e^{1j\phi_1} e^{2j(\phi_2+\phi_3)} - \\ & C_1 C_2^2 C_3^2 S_0 S_4 e^{2j(\phi_2+\phi_3)} + C_0 C_1^2 C_2^2 C_3 C_4 e^{1j\phi_1} e^{2j(\phi_2+\phi_3)} \end{aligned} \tag{S11}$$

$$a_0 = e^{1j\phi_1} e^{2j(\phi_2+\phi_3)} e^{2j(\phi_2+\phi_3} \tag{S12}$$

$$a_1 = -C_1 C_2 e^{1j\phi_1} e^{2j\phi_3} e^{2j(\phi_2+\phi_3)} - C_2 C_3 e^{1j\phi_1} e^{2j\phi_2} e^{2j(\phi_2+\phi_3)} \tag{S13}$$

$$a_2 = C_1 C_3 e^{1j\phi_1} e^{2j(\phi_2+\phi_3)} \tag{S14}$$

2. COMPARISON WITH DOUBLE RING LOADED MZI WITHOUT COUPLING BETWEEN THE RINGS

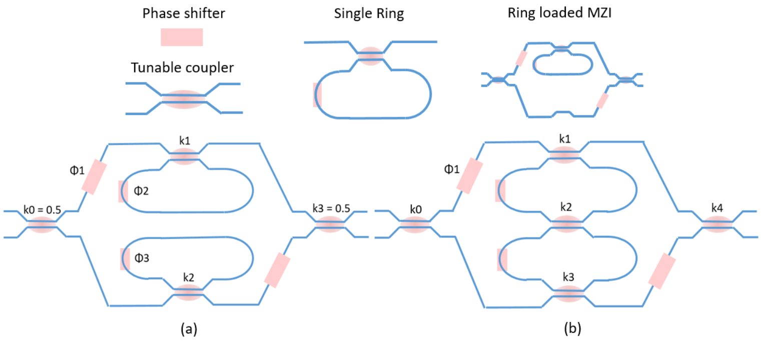

Fig. S1. Schematic drawing of the classical ring loaded MZI is shown in (a); the schematic drawing of coupled ring loaded MZI is shown in (b). The main difference between our design given in (b) and the classical double ring loaded MZI given in (a) is that the two rings are connected with a tunable coupler. It is obvious that the design in (b) could be configured to an MZI with delay length of twice the ring circumference, which is not possible with the design in (a). The phase shifters are shown as a pink box, and the tunable coupler is shown as a normal DC with a pink dot in the middle, the single add drop ring is constructed by connecting the input and output port of the tunable coupler on the same side. The input and output tunable coupler of the design in (b) is realized by a broadband balanced MZI [1] on the actual chip.

In this section, we will compare the pole-zero diagram of the classical ring loaded MZI and of our design. We demonstrate that with the same loss characteristics of the couplers, both design could cover the same area of the unit circle in the z-plane, and thus both have the potential to exactly realize second-order bandpass filter.

The ring loaded MZI in Fig. S1(a) is a complex filter where its a_i and b_i coefficients are complex numbers, however the coupled ring loaded MZI in Fig. S1(b) can be configured both as a real filter and as a complex filter.

In the following, we would configure our tunable filter design into the double ring loaded MZI as shown in Fig. S1(b) and ring loaded MZI as shown in Fig. S1(a) separately.

The coupled ring loaded MZI in Fig. S1(b) has 6 free parameters, which are k_0, k_1, k_2, k_3, k_4 and ϕ_1. In simulation we directly modify k_i, but in the experiment the coupling factors k_i are realized using a balanced MZI with a phase shifter in each arm, and thus the tuning parameter becomes another phase shift. The coupled ring loaded MZI in Fig. S1(b) can be configured to be a real filter where its coefficients are real numbers. In order to force the coefficients to be real, the phase shifters inside the rings need to be set to either zero (configuration I) or π (configuration II). These two situations are equivalent with each other in the spectrum response, we will discuss these two situations in Fig. S2.

The ring loaded MZI in Fig. S1(a) can also be a real filter, but then its configurability is limited. To implement a generic second-order bandpass filter, it needs to be configured as a complex filter where its a_i and b_i coefficients are complex numbers. The classical ring loaded MZI has 5 free parameters in simulation, which are k_1, k_2, ϕ_1, ϕ_2, ϕ_3, where the input and output couplers are set to $k_0 = k_3 = 0.5$.This architecture is proposed for realizing a bandpass filter with two all-pass filters in [2]. For most cases, a symmetric spectral response is desired, which requires $k_1 = k_2$ and $\phi_1 = -\phi_2$.

The filter synthesis equations have been accordingly rewritten for the ring loaded MZI in Fig. S1(a), the equations Eq. (S4) - Eq. (S14) for the ring loaded MZI could be rewritten by setting the coupling k_2 to 0, thus $C_2 = 0$ and $S_2 = 1$. Then we compare the two types of filter in simulation. We will illustrate the programmability space of the two filter types using the Latin Hypercube sampling (LHS) method. LHS is a statistical method for generating a near-random sample of parameter values from a multidimensional distribution. In our following analysis, we use this method to explore the relationship between the parameter space and the distribution of the filter's poles and zeros in the z-plane.

For both cases, 500 sampling points are used. We plot the pole-zero map of both structures in Fig. S2. From Fig. S2, we can see that the poles of Fig. S1(a) are homogeneously distributed in the z-plane. The poles within the same pair are not necessarily symmetrically positioned with respect to the Real (x) axis. The zeros are mostly located in the unit circle. The poles for configuration I ($\phi_1 = 0$) of Fig. S1(b) are homogeneously distributed in the right half-plane of the unit circle, while the zeros are scattered homogeneously, also in the right half of the z-plane. The poles of configuration II ($\phi_1 = \pi$) of Fig. S1(b) are homogeneously distributed in the left half-plane of the unit circle, the zeros are scattered homogeneously also in the left-half of the z plane.

These two designs (coupled ring-loaded MZI and ring-loaded MZI) show full configurability. In the following, we would show how to get the desired coupling values for a certain filter with both complex and real filter coefficients. We will use three examples to show that these two designs are equivalent with each other, however one may perform better than the other in certain cases.

The following target function is used for optimizing a complex filter:

$$\begin{aligned} sum_{ab} = ((a_0 \cdot a_0^* - {a'_0}^2)^2 + (a_1 \cdot a_1^* - {a'_1}^2)^2 + (a_2 \cdot a_2^* - {a'_2}^2)^2 + \\ (b_0 \cdot b_0^* - {b'_0}^2)^2 + (b_1 \cdot b_1^* - {b'_1}^2)^2 + (b_2 \cdot b_2^* - {b'_2}^2)^2)^{1/2} \end{aligned} \tag{S15}$$

Likewise, the target function for real filter is:

$$\begin{aligned} sum_{ab} = ((\overset{2}{a}_0 - {a'_0}^2)^2 + (\overset{2}{a}_1 - {a'_1}^2)^2 + (\overset{2}{a}_2 - {a'_2}^2)^2 + \\ (\overset{2}{b}_0 - {b'_0}^2)^2 + (\overset{2}{b}_1 - {b'_1}^2)^2 + (\overset{2}{b}_2 - {b'_2}^2)^2)^{1/2} \end{aligned} \tag{S16}$$

Let's build a first example filter with a second-order elliptical low-pass filter with 1 dB in-band ripple, 20 dB extinction ratio and the normalized edge frequencies of 0.1 π rad/sample. The a_i and b_i coefficients for such filter can be calculated with Matlab:

$$b_1 = [0.1055, -0.1245, 0.1055] a_1 = [1.0000, -1.6293, 0.7264] \tag{S17}$$

We will now compare the filter responses of configuration I of Fig. S1(b) and complex filter in Fig. S1(a). After we apply the synthesis algorithm as described before, the pole-zero plot and

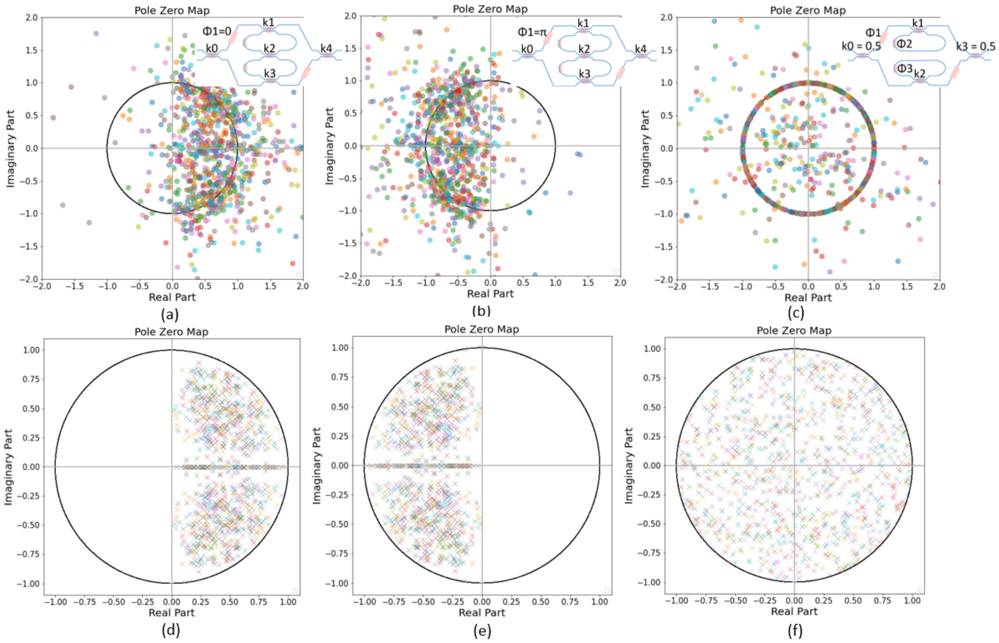

Fig. S2. Pole and zero distribution in the z-plane for a Latin Hypercube sampling of the tuning coefficients of both types of filters. The zeros of Fig. S1(b) configuration I ($\phi_1 = 0$) are plotted in (a), the poles of Fig. S1(b) configuration I are plotted in (e).The zeros of Fig. S1(b) configuration II ($\phi_1 = \pi$) are plotted in (b), the poles of Fig. S1(b) configuration II are plotted in (e).The zeros of Fig. S1(a) are plotted in (c), the poles of Fig. S1(a) are plotted in (f).

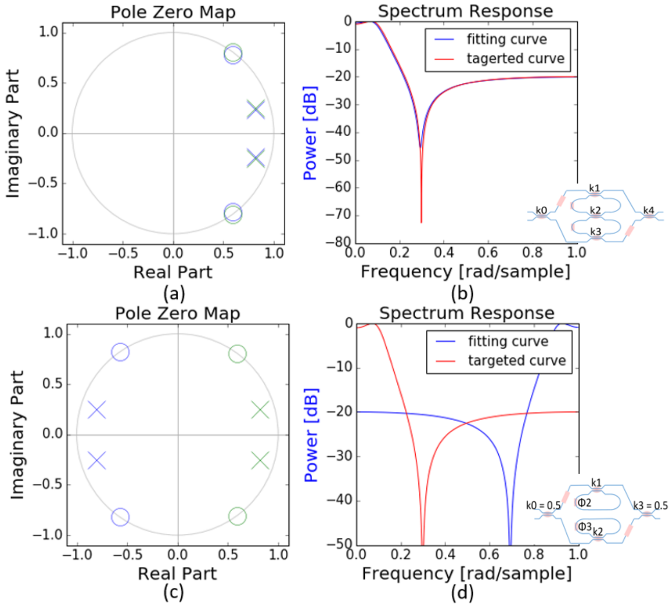

Fig. S3. The first example filter has been synthesized using both the classical and the coupled ring-loaded MZI. (a) and (c) are the pole-zero diagram of the synthesized filter, (b) and (d) are the spectrum responses of the synthesized filter compared to the targeted curve. (a) and (b) correspond to the coupled ring-loaded MZI of Fig. S1(b), and (c) and (d) corresponds to the classical ring-loaded MZI of Fig. S1(a).

the spectrum response are shown in Fig. S3 for both filter configurations.The fitting curve is the synthesized result and the targeted curve is the ideal elliptical filter response we would like to

ellip(2,1,20,0.1,’low’),	b1=[1.055,-0.1245,0.1055],a1=[1,-1.6293,0.7264]
our design	ring loaded MZI
$k_0 = k_4 = 0.438, k_1 = k_3 = 0.2736$<br>$k_2 = 0.0869, phi_1 = 0$	$k_0 = k_3 = 0.5, k_1 = k_2 = 0.2736$<br>$phi_1 = -0.0790pi, phi_2 = -phi_3 = 0.4525pi$
$b = [(0.1055, -0.1245, 0.1013)]$	$b = [(-0.0131 + 0.1047j), (-0.0153 - 0.1234j),$<br>$(-0.0131 - 0.1047j)]$
$a = [1, -1.6302, 0.7192]$	$a = [(0.9694 - 0.2456j), (1.5794 - 0.4001j),$<br>$(0.7042 - 0.1784j)]$

Table S1. Parameters For Fig. S3

ellip(2,1,20,0.1,’low’)	b1=[1.055,-0.1245,0.1055],a1=[1,-1.6293,0.7264]
solution 1	$k_0 = k_4 = 0.438, k_1 = k_3 = 0.2736, k_2 = 0.0869, phi_1 = 0$
solution 2	$k_0 = 0.9847, k_1 = k_3 = 0.2736, k_2 = 0.0864, k_4 = 0, phi_1 = 0$
solution 3	$k_0 = 0.6625, k_1 = k_3 = 0.2736, k_2 = 0.0869, k_4 = 0.2263, phi_1 = 0$
solution 4	$k_0 = 0.4270, k_1 = 0.2614, k_2 = 0.0858, k_3 = 0.2865, k_4 = 0.4494, phi_1 = 0$

Table S2. 4 solutions for Fig. S3(b)

achieve.

The corresponding parameters for Fig. S3 are given in Table S1.According to our experiments, we found out that for normal filters such as elliptical filter, the structure is actually symmetrical, thus, certain constrains could be applied for the two designs when we try to solve for the couplings and phases. For our design shown in Fig. S1(a), the constrains are $k_0 = k_4, k_1 = k_3$ and $phi_1 = 0$. For the ring loaded MZI, the constrains are $k_0 = k_3 = 0.5, k_1 = k_2$ and $phi_2 = -phi_3$. There are more solutions for our design when a given elliptical filter is synthesized, four solutions for the same elliptical filter in example one is shown in Table S2.As we can see, solution 1 is the most symmetrical result, and solution 2 will simplify our design since no output coupler is needed. Filter designer might select the most stable design or the one with less wavelength dispersion.

The pole-zero plot and corresponding transfer function for the coupled ring-loaded MZI are shown in Fig. S3(a) and Fig. S3(b). In Fig. S3(a), the target pole and zero positions overlap with the fitted ones, and we see a perfect correspondence for their spectrum response. The pole-zero plot and corresponding transfer function for the conventional ring loaded MZI are shown in Fig. S3(c-d). In Fig. S3(c), the target poles and zeros are in the right half of the z plane, while the fitted one is in the left half plane. This corresponds to a shift of the spectral response with half a free spectral range (FSR), or a mirroring on the Nyquist frequency axis. Since the spectrum response is repeated with the FSR, so the final spectrum response of the targeted result and the fitted result show no difference with each other.

Now we consider a second filter example, let’s construct a Chebyshev type II filter cheby2(2 ,20, 0.9, ’low’). The a_i and b_i coefficients are as following:

$$b_1 = [0.6326, 1.2339, 0.6326] a_1 = [1.0000, 1.0928, 0.4063] \tag{S18}$$

The pole-zero diagram and the corresponding spectrum response of the coupled ring filter in configuration II ($\phi_1 = \pi$) are shown in Fig. S4(a-b).The pole-zero diagram and the corresponding spectrum response of the conventional ring loaded MZI is shown in Fig. S4(c-d). The corresponding parameters for Fig. S4 is given in Table S3.

The two circuits perform equivalently. The spectrum responses are the same if we would like to systhesize a given bandpass filter, and the only difference would lie in the configuration of the filter and its corresponding a_i and b_i coefficients.

For the third example, the a_i and b_i coefficients are not calculated from any bandpass filters. We try to set the following values to our design, $k_0 = 0.6$, $k_1 = 0.9$, $k_2 = 0.5$, $k_3 = 0.1$, $k_4 = 0.4$

cheby2(2,20,0.9,'low'),	b1=[0.6326,1.2339,0.6326],a1=[1,1.0928,0.4063]
our design	ring loaded MZI
$k_0 = k4 = 0.9962, k_1 = k_3 = 0.5937$<br>$k_2 = 0.2652, phi_1 = 0$	$k_0 = k_3 = 0.5, k_1 = k_2 = 0.5730$<br>$phi_1 = 0.8388pi, phi_2 = -phi_3 = 0.4077pi$
$b = [(-0.6326, 1.2339, -0.6326)]$	$b = [(-0.6124 + 0.1585j), (-1.1945 + 0.3091j),$<br>$(-0.6124 + 0.1585j)]$
$a = [1, -1.0928, 0.4108]$	$a = [(-0.8745 + 0.4851j), (-0.3734 + 0.2071j),$<br>$(-0.3734 + 0.2071j)]$

Table S3. Parameters For Fig. S4

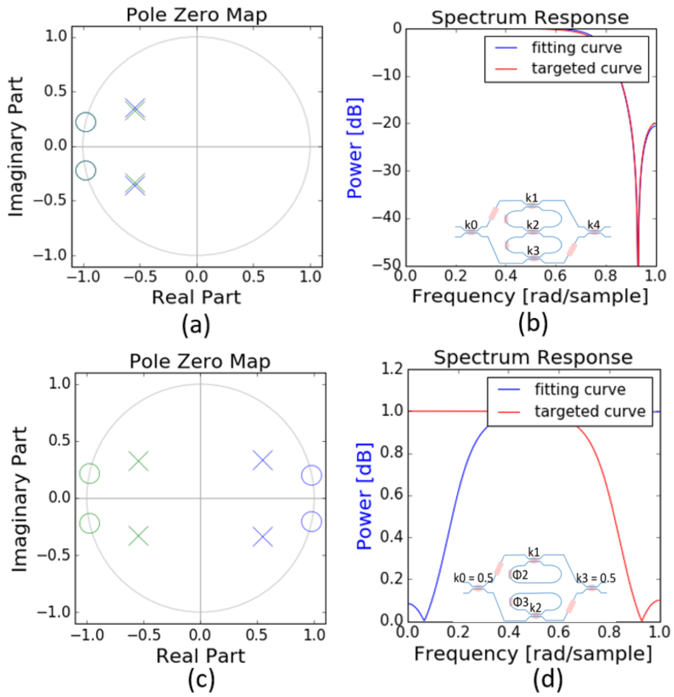

Fig. S4. The second example filter has been synthesized by both the classical and the coupled ring-loaded MZI. (a) and (c) represent the pole-zero diagrams of the synthesized filters, while (b) and (d) show their spectrum responses compared with the targeted curve. (a) and (b) corresponds to structure of the coupled ring-loaded MZI of Fig. S1(b), and (c) and (d) corresponds to the classical ring-loaded MZI of Fig. S1(a).

and $\phi_1 = \pi$. The a_i and b_i coefficients are calculated, and then we try to fit the ring loaded MZI with the calculated coefficients, the a_i and b_i values are given in Eq. S19:

$$b_1 = [-0.6197, 0.8582, -0.6197] a_1 = [-1.0000, 0.8944, -0.3000] \quad \text{(S19)}$$

The pole-zero diagram and the corresponding spectrum response of the coupled ring-loaded MZI in configuration II are shown in Fig. S5(a-b).The pole-zero diagram and the corresponding spectrum response of ring loaded MZI is shown in Fig. S5(c-d). The parameters for Fig. S5 are given in Table S4. In this case, the fitted curve and the targeted curve in Fig. S5(d) did not overlap with each other very well, which indicates that the ring loaded MZI could not be configured to match the double ring loaded MZI. The double ring loaded MZI thus is a better candidate for a fully programmable filter. This is important when considering uses other than bandpass filters, such as arbitrary waveform generation.

	b1=[-0.6197,0.8582,-0.6197],a1=[-1,0.8944,-0.3]
our design	ring loaded MZI
$k_0 = 0.6, k_1 = 0.9, k_2 = 0.5$ $k_3 = 0.1, k_4 = 0.4, phi_1 = pi$	$k_0 = k_3 = 0.5, k_1 = k_2 = 0.5730$ $phi_1 = 0.8388pi, phi_2 = -phi_3 = 0.4077pi$
$b = [(-0.6197, 0.8582, -0.6197)]$	$b = [(-0.6021 + 0.1467j), (-0.8339 + 0.2031j),$ $(-0.6021 + 0.1467j)]$
$a = [-1, 0.8944, -0.3]$	$a = [(-0.8880 + 0.4600j), (-0.7942 + 0.4113j),$ $(-0.3612 + 0.1871j)]$

Table S4. Parameters For Fig. S5

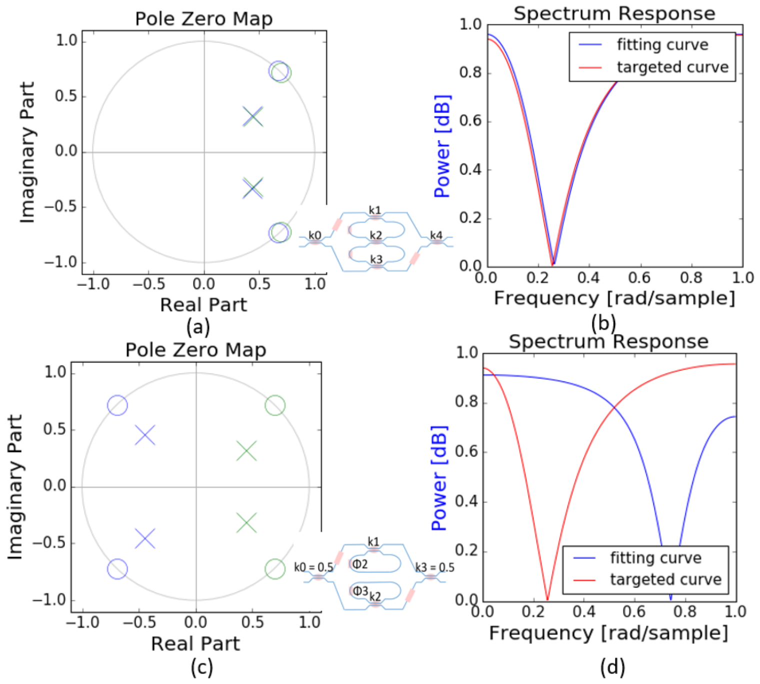

Fig. S5. The third example filter has been synthesized for both filter circuits. (a) and (c) are the pole-zero diagrams of the synthesized filters, while (b) and (d) show their spectrum responses as well as the targeted response. (a) and (b) correspond to the coupled ring-loaded MZI of Fig. S1(b), while (c) and (d) correspond to the classical ring-loaded MZI of Fig. S1(a).

As we can see from the three examples, the a_i and b_i coefficients in our design are always real numbers. Actually if the ϕ_1 is set not be 0 or π, the a_i and b_i coefficients would also be complex number for our design. If we try to fit such complex a_i and b_i by the given ring loaded MZI, we can not get a good fit, either. Such situation is very similar to the third example.

3. CASCADE FOR HIGHER ORDER FILTER

The roll-off (measured in dB/decade) reduces by 20 dB/decade per filter order increment. Thus, if a second-order filter function has a roll-off of 20 dB/decade, the fourth-order filter would have a roll-off of 60 dB/decade. This results in a more ideal amplitude frequency response. On the other hand, higher-order filters are complex to configure and design, and they are used only when a sharp transition between the passband and stopband is required.

We take a fourth-order Chebyshev type II filter with 30 dB stop band and 0.5π rad/dB as an example. The power response of the Chebyshev filter will have a 60 dB extinction ratio. The

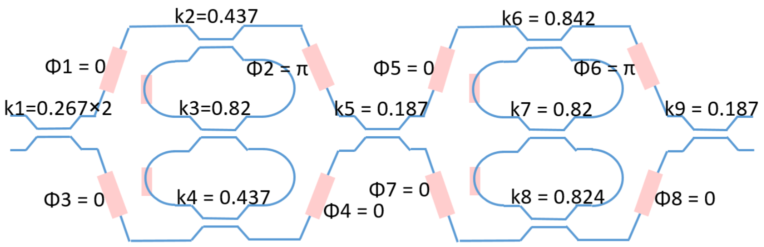

Fig. S6. Schematic of a two stage filter and the corresponding coupling values and phase response for a fourth-order Chebyshev type II filter with 30 dB stop band and 0.5π rad/dB.

transfer function of such a filter is given by:

$$H_z = \frac{0.0996 + 0.164 \cdot z^{-1} + 0.2226 \cdot z^{-2} + 0.164z^{-3} + 0.0996 \cdot z^{-4}}{1 - 0.9055z^{-1} + 0.8128 \cdot z^{-2} - 0.2016 \cdot z^{-3} + 0.0442 \cdot z^{-4}} \tag{S20}$$

Eq. S20 can be decomposed to two second-order filters, $H_1(z)$ and $H_2(z)$. The corresponding second-order transfer functions are:

$$H_1(z) = \frac{0.35 + 0.0553 \cdot z^{-1} + 0.35 \cdot z^{-2}}{1 + 0.6362 \cdot z^{-1} + 0.5629 \cdot z^{-2}} \quad H_2(z) = \frac{0.25 + 0.3723 \cdot z^{-1} + 0.25 \cdot z^{-2}}{1 - 0.2694 \cdot z^{-1} + 0.0785 \cdot z^{-2}} \tag{S21}$$

Then we use least-square fitting to find the corresponding coupling value for the each subcircuit. Fig. S6 is the schematic drawing of a two-stage filter and the corresponding coupling values and phase response from the least square fitting.

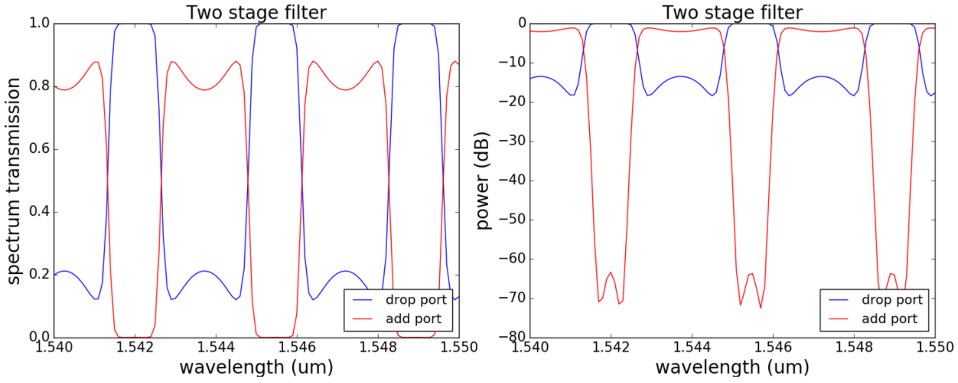

Fig. S7. Spectrum response for two-stage filter configured for Chebyshev type II response

We then build the Caphe circuit model for the circuit in Fig. S6 and we can see the corresponding spectrum response in Fig. S7. This shows that the designed filter can reach an extinction ratio of around 60 dB, which meets our requirement.

4. PROGRAMMABLE FILTER DESIGN

Programmable photonic circuits have been well studied in recent years [3], and there have been several demonstrations to achieve a programmable optical filter [4]. The reconfigurability is desired in many applications such as optical interleavers, spectral slicers, or filtering and equalization circuits for microwave photonics [5]. The ability to change its response according to the requirements would on one hand lower the price of the communication channel, on the other hand, it would allow the software to correct or adjust itself with feedback , which is beneficial to the signal performance.

For a programmable filter, we need to be able to adjust the different phases and coupling ratios in our filter circuit. Instead of passive directional couplers, we can use a tunable coupler, as

shown in Fig. S8(a). In this work, the tunable coupler is composed of a balanced Mach-Zehnder interferometer, but other types of tunable coupler designs have also been proposed to compensate for fabrication variation or dispersion [1].

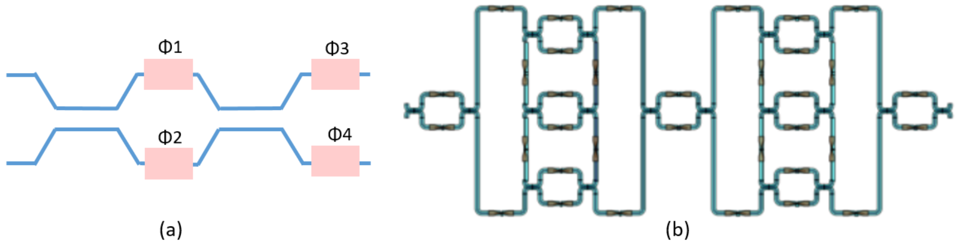

Fig. S8. (a) is the schematic drawing of a tunable coupler, the pink box denotes the phase shifter, the input and output coupler are 50/50 directional couler. (b) is the schematic drawing of a programmable two stage filter. The couplers in Fig. S6 have been replaced by tunable coupler shown in (a).

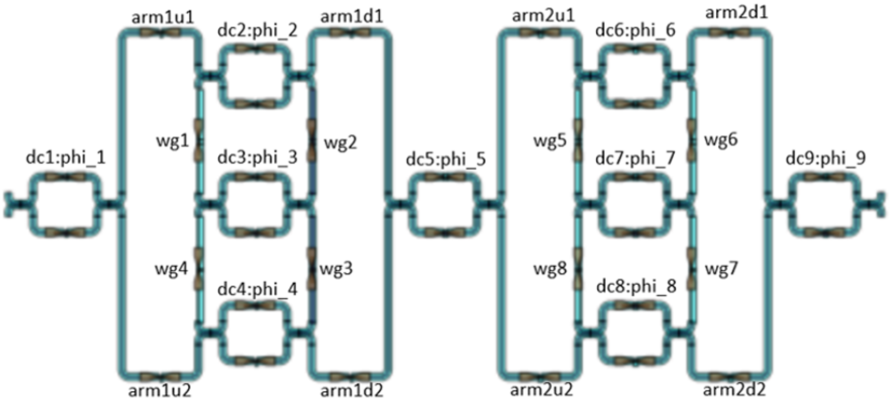

Fig. S9. Schematic drawing of the implementation of a two-stage programmable filter based on the coupled ring-loaded MZI.

The phases ϕ_1 or ϕ_2 are used to adjust the power coupling. This also introduces a phase shift $(\phi_1 + \phi_2)/2$ at the outputs. So in order to maintain the same phase response as a static directional coupler, we need to use ϕ_3 and ϕ_4 to compensate this additional phase change induced by ϕ_1 and/or ϕ_2. This need for compensation complicates the control of the entire circuit somewhat. If we set the phase shifter of the upper arm of the MZI to be $\phi_1 = x$, and lower arm of the phase shifter to be zero, in order to induce no phase change compared to a passive coupler, then, ϕ_3 and ϕ_4 should have the following value, $\phi_3 = \phi_4 = 2m\pi - x/2$, with m an integer number. For phase shifters that can induce both a positive and negative phase shift, m is preferably zero, but for phase shifters that can only operate in a single direction, such as heater-based phase shifters, $m = 1$.

We replaced all the couplers in Fig. S6 with the tunable coupler in Fig. S8(a), and built a circuit model for the two-stage filter in Fig. S9(b). We then translated the desired coupling in Fig. S6(b) to the corresponding phase values in Fig. S9(a), including the compensation phases. These parameters of phases are shown in Table S5. The spectrum response simulated with the Caphe model of Fig. S9(a) is then shown in Fig. S10.

5. ESTIMATION OF Q FACTOR

In this section, we will have an estimation of the Q factor [6]. We assume the loss for the tunable coupler is 0.12 dB, n_g is 4.3, the loss for the phase shifter is around 0.02 dB. The resonance

dc:phase	Phase	wg:phase		Arm:phase	
dc1: ϕ_1	$\phi_1 = 0.5275\pi$	wg1:ϕ_1	0	Arm1u1:ϕ_1	-$\phi_1/2$
dc2: ϕ_2	$\phi_2 = 0.5402\pi$	wg2:ϕ_2	-(ϕ_2 +ϕ_3)/2	Arm1u2:ϕ_2	-$\phi_1/2$
dc3: ϕ_3	$\phi_3 = 0.279\pi$	wg3:ϕ_3	0	Arm1u2:ϕ_2	-$\phi_2/2 + \pi$
dc4: ϕ_4	$\phi_4 = \phi_2$	wg4:ϕ_4	-($\phi_4 + \phi_3$)/2	Arm1d2:ϕ_4	-$\phi_4/2$
dc5: ϕ_5	$\phi_5 = 0.5\pi$	wg5:ϕ_5	0	Arm2u1:ϕ_5	-$\phi_5/2$
dc6: ϕ_6	$\phi_6 = 0.26\pi$	wg6:ϕ_6	-($\phi_6 + \phi_7$)/2	Arm2u2:ϕ_6	-$\phi_5/2$
dc7: ϕ_7	$\phi_7 = 0.279\pi$	wg7:ϕ_7	0	Arm2d1:ϕ_7	-$\phi_6/2 + \pi$
dc8: ϕ_8	$\phi_8 = 0.2755\pi$	wg8:ϕ_8	-($\phi_8 + \phi_7$)/2	Arm2d2:ϕ_8	-$\phi_8/2$
dc9: ϕ_9	$\phi_9 = \phi_5$				

Table S5. Parameters For Fig. S9

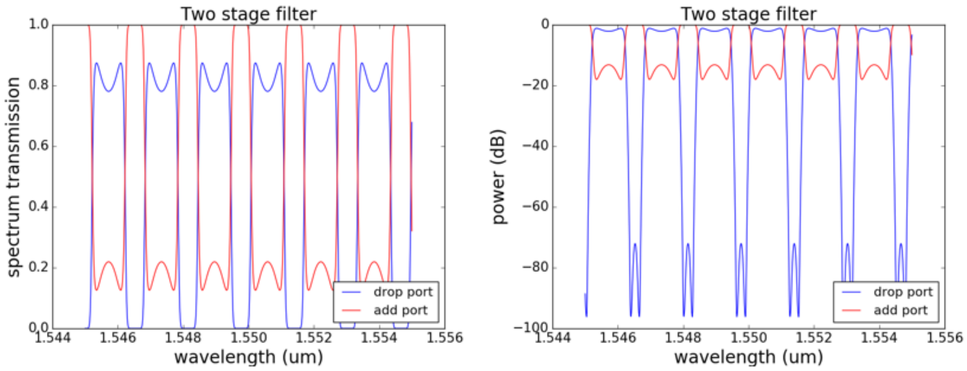

Fig. S10. Simulation result for the Butterworth filter using the two-stage design with the corresponding caphe model

wavelength is 1.55 *um*, and the round trip length L of the ring is calculated according to the FSR measured and it is 712 *um*, the length of the tunable coupler is 173.4 um and the length of the heater is 50 *um*. The waveguide loss is estimated to be 1.3 dB/cm. r is the critical coupling and g is the amplitude attenuation coefficient.

The loss of power of the single ring is composed of waveguide loss, loss for two tunable couplers and the loss of the phase shifter, the roundtrip loss of the single ring is 0.300976 *dB*.

$$Loss_{dB} = -10\log_{10}(P_{out}/P_{in}) = -20\log_{10}(e^{-gL}) = 20gL \cdot \log_{10} e \tag{S22}$$

The Q factor of the single ring is calculated by the following equation:

$$Q = -\pi \frac{L_{ring}}{\lambda_{res} \cdot (\ln r - g \cdot L_{ring})} \tag{S23}$$

The Q at the critical coupling (i.e. $r = g$) is estimated to be 18184.

6. TOLERANCE ANALYSIS

In experiments, the dispersion of the building blocks, as well as the fabrication variation and thermal cross-talk need to be considered, as they move the filter away form its nominal operating point and complicate the configuration and control. Fig. S11 is a Monte-Carlo simulation performed for two filter designs in Fig. S6 for a given filter response, and it is hard to tell which filter is better in terms of fabrication variation. It is certain, however, that the phase shifters inside the ring contribute the most significantly to the deformation of the spectrum response.

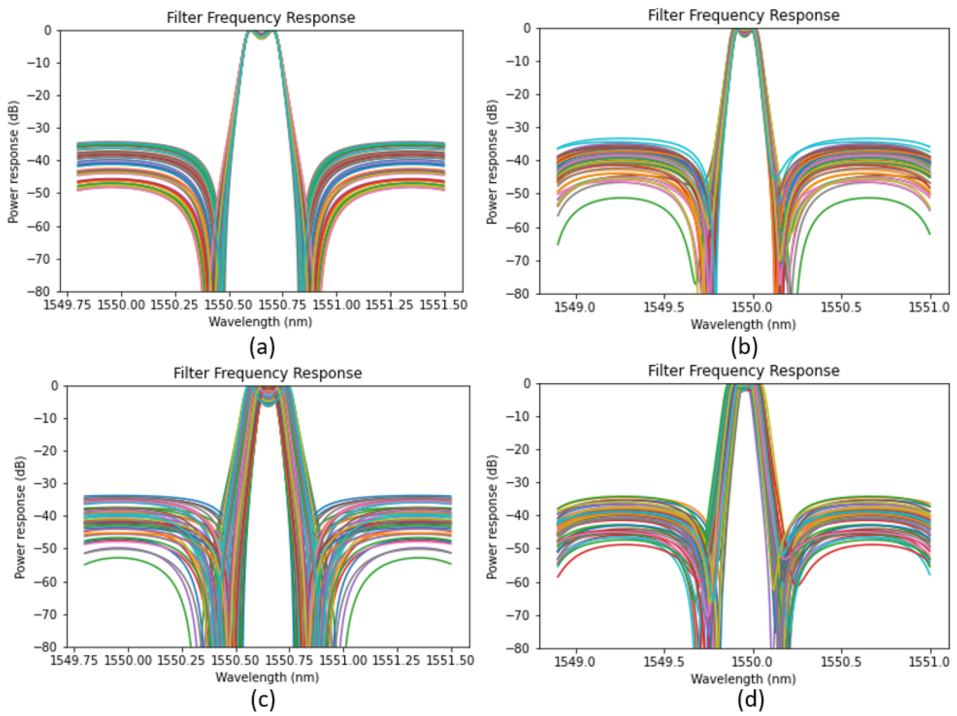

Fig. S11. Monte-Carlo simulation for two filter configurations in Fig. S6, (a) and (c) are for the classical ring-loaded MZI design, (b) and (d) are for the coupled ring-loaded MZI design. For (a) and (b), 5% parameter variation is applied to all the parameters except for the phases in the rings. For (c) and (d), all the parameters including the ring phase shifters) have a 5% variation in the Monte-Carlo simulation.

REFERENCES

1. M. Wang, A. Ribero, Y. Xing, and W. Bogaerts, “Tolerant, broadband tunable 2× 2 coupler circuit,” Opt. express **28**, 5555–5566 (2020).
2. C. Madsen, “Efficient architectures for exactly realizing optical filters with optimum bandpass designs,” IEEE Photonics Technol. Lett. **10**, 1136–1138 (1998).
3. W. Bogaerts, D. Pérez, J. Capmany, D. A. B. Miller, J. Poon, D. Englund, F. Morichetti, and A. Melloni, “Programmable photonic circuits,” Nature **586**, 1476–4687 (2020).
4. G. Choo, S. Cai, B. Wang, C. K. Madsen, K. Entesari, and S. Palermo, “Automatic monitor-based tuning of reconfigurable silicon photonic apf-based pole/zero filters,” J. Light. Technol. **36**, 1899–1911 (2018).
5. D. Pérez, A. López, P. DasMahapatra, and J. Capmany, “Multipurpose self-configuration of programmable photonic circuits,” Nat. Commun. **6359**, 2041–1723 (2020).
6. W. Bogaerts, P. De Heyn, T. Van Vaerenbergh, K. De Vos, S. Kumar Selvaraja, T. Claes, P. Dumon, P. Bienstman, D. Van Thourhout, and R. Baets, “Silicon microring resonators,” Laser & Photonics Rev. **6**, 47–73 (2012).
